# Propagation of kinetic uncertainties through a canonical topology of the TLR4 signaling network in different regions of biochemical reaction space

**DOI:** 10.1186/1742-4682-7-7

**Published:** 2010-03-15

**Authors:** Jayson Gutiérrez, Georges St Laurent, Silvio Urcuqui-Inchima

**Affiliations:** 1 Grupo de Física y Astrofísica Computacional (FACom), Instituto de Física, Universidad de Antioquia, Medellin, Colombia; 2 Department of Molecular Biology, Cell Biology, and Biochemistry, Brown University, Providence, RI 02912, USA; 3 Grupo de Inmunovirología, SIU, Universidad de Antioquia, Medellin, Colombia

## Abstract

**Background:**

Signal transduction networks represent the information processing systems that dictate which dynamical regimes of biochemical activity can be accessible to a cell under certain circumstances. One of the major concerns in molecular systems biology is centered on the elucidation of the robustness properties and information processing capabilities of signal transduction networks. Achieving this goal requires the establishment of causal relations between the design principle of biochemical reaction systems and their emergent dynamical behaviors.

**Methods:**

In this study, efforts were focused in the construction of a relatively well informed, deterministic, non-linear dynamic model, accounting for reaction mechanisms grounded on standard mass action and Hill saturation kinetics, of the canonical reaction topology underlying Toll-like receptor 4 (TLR4)-mediated signaling events. This signaling mechanism has been shown to be deployed in macrophages during a relatively short time window in response to lypopolysaccharyde (LPS) stimulation, which leads to a rapidly mounted innate immune response. An extensive computational exploration of the biochemical reaction space inhabited by this signal transduction network was performed via local and global perturbation strategies. Importantly, a broad spectrum of biologically plausible dynamical regimes accessible to the network in widely scattered regions of parameter space was reconstructed computationally. Additionally, experimentally reported transcriptional readouts of target pro-inflammatory genes, which are actively modulated by the network in response to LPS stimulation, were also simulated. This was done with the main goal of carrying out an unbiased statistical assessment of the intrinsic robustness properties of this canonical reaction topology.

**Results:**

Our simulation results provide convincing numerical evidence supporting the idea that a canonical reaction mechanism of the TLR4 signaling network is capable of performing information processing in a robust manner, a functional property that is independent of the signaling task required to be executed. Nevertheless, it was found that the robust performance of the network is not solely determined by its design principle (topology), but this may be heavily dependent on the network's current position in biochemical reaction space. Ultimately, our results enabled us the identification of key rate limiting steps which most effectively control the performance of the system under diverse dynamical regimes.

**Conclusions:**

Overall, our *in silico *study suggests that biologically relevant and non-intuitive aspects on the general behavior of a complex biomolecular network can be elucidated only when taking into account a wide spectrum of dynamical regimes attainable by the system. Most importantly, this strategy provides the means for a suitable assessment of the inherent variational constraints imposed by the structure of the system when systematically probing its parameter space.

## Background

Normal and abnormal cellular states represent macroscopic behaviors emerging from intricate dynamical patterns (either transient or stationary) of biochemical activity. These are sustained by a complex web of reaction mechanisms that play the role of information processing systems, generically referred to as signal transduction networks [1-3]. In other words, these networks represent the dynamical systems that instruct cells to enter into specific regimes of biochemical activity, which ultimately determine the universe of functional states accessible to the cell, such as differentiation, apoptosis, cell division, etc. [1-3]. Operatively, functional regimes of biochemical activity within a cell are basically accomplished via direct protein-protein interactions and enzyme-catalyzed reactions (i.e. phosphorylation, RNA synthesis, etc.) triggered in response to either internal or external stimuli [3,4].

The spectrum of functionalities that a signal transduction network can potentially perform is inherently constrained by its design principle [5,6], which encapsulates a series of aggregated components involving diverse regulatory schemes and biochemical reaction rules modulated quantitatively via internal reaction parameters. This structure-function puzzle has motivated considerable research efforts in the last decade aimed at elucidating possible mechanistic bases of fundamental emergent properties such as robustness, evolvability and epistasis, of highly-modular regulatory systems [7-13]. Importantly, the investigation of the robustness properties of a signal transduction network requires heavy emphasis to be made on two fundamental aspects of the underlying reaction mechanism: an observable/quantifiable dynamical feature (either transient or stationary) of the system, and one or several perturbable parameters directly or indirectly involved in the development of the system's feature being studied. For instance, important quantitative dynamical features of signal transduction networks have been proposed as suitable targets for assessing their robustness properties in the face of random changes in internal reaction parameters [14,15]. Sources of perturbations impinging upon such parameters may stem from environmental vicissitudes (temperature, pH, etc.), genotypic variation or intrinsic fluctuations (molecular noise) [16,17].

Recently, several computational studies have yielded interesting numerical evidence supporting the idea that the robustness properties of highly-dimensional biochemical reaction networks may be strongly dependent on three fundamental aspects: *i*) the reaction topology (network architecture) [7-9], *ii*) the system's current position in parameter space [18-20], and *iii*) the dynamic nature of the trajectories displayed by the reaction species involved [13,20-22]. The robustness properties of a biomolecular network are typically assessed by means of standard sensitivity analysis-based approaches implementing both local and global perturbation methods [18,23-27]. Robustness is usually assessed with respect to either observable or hypothetical stationary states and transient dynamics of just few reaction species in the network [24,28,29]. However, a complementary quantitative approach to studying the robustness properties, as well as information processing capabilities, of a complex reaction network should provide the means for assessing the extent to which the full dynamical behavior of the system is reproducible under, for example, kinetic uncertainties. This is because a reaction network may be coupled dynamically in unexpected ways to other important subsystems not included in the model [11,30], whereby biochemical information exchange among cellular processes can take place in parallel. Under these considerations, we thus believe that general properties of a canonical biomolecular network could be revealed under the following methodological strategies. Firstly, a large ensemble of disparate, but biologically plausible dynamical trajectories attainable by the network should be tested for general robustness properties in the face of random perturbations impinging upon the whole set of reaction parameters; that is to say, the overall robust performance of the network should be evaluated in widely scattered regions of its accessible parameter space. Secondly, the reproducibility of particular ouputs (i.e. experimentally reported wild-type transcriptional readouts) should be assessed in different regions of the accessible parameter space via both local and global perturbation strategies. Addressing these points would pave the way to gaining general insight into systems-level features of the complex reaction mechanisms endowing the cells with the potential to reach a wide spectrum of robust behaviors.

In this study, efforts were focused on a comprehensive and unbiased statistical assessment of the robustness properties and information processing capabilities of a canonical reaction topology underlying TLR4-mediated signaling events. This signaling network is temporally deployed in inflammatory cells (i.e. macrophages) in response to external stimuli. We constructed a deterministic, non-linear dynamic model of this reaction topology, using an informational basis retrieved from a series of previous computational studies and review papers providing important clues about mechanistic reaction steps involved in the process (see the Results and Discussion section below). We adopted this signaling network as our model system mainly because this functional module plays a crucial role in the development of innate immune cellular responses ([31-37]). For instance, Toll-like receptors recognize conserved pathogen-associated molecular patterns such as lipopolysaccharide (LPS), which results in the triggering of both microbial clearance and the induction of immunoregulatory chemokines and cytokines. Here, we centered our attention specifically on the immediate cellular response, in macrophages, triggered by the rapid activation of the canonical MyD88-dependent and TRIF-dependent reaction cascades upon LPS binding to TLR4. We probed the robustness properties and information processing capabilities of this canonical network in different points distributed across diverse regions of the biochemical reaction space. Importantly, the behavior of the network in a given region of the biochemical reaction space was selected so that it was congruent with a hypothetical, but biologically plausible dynamical regime of molecular activity (see below). Global (non-orthogonal) and local (orthogonal) perturbation strategies were implemented as a means of systematically exploring the biochemical reaction space inhabited by the network. Critically, reaction parameters were subjected to random perturbations without *a priori *knowledge on their relative importance for the network in the accomplishment of a given signaling task. Our extensive numerical analyses permitted us the identification of global and particular variational constraints in the network. This was achieved by means of a detailed characterization of some statistical regularities on the dynamical performance of the system under kinetic uncertainties (i.e. random fluctuations in internal reaction parameters). Overall, our simulation results provide convincing numerical evidence supporting the following idea: a canonical reaction mechanism underlying TLR4-mediated signaling events is endowed with the intrinsic capacity to perform information processing in a robust manner, which is remarkably independent of the signaling task required to be executed. Nevertheless, our statistical analysis indicate that the robust performance of the network is not solely determined by its architecture (topology), but this may be strongly conditioned by the network's current position in biochemical reaction space. Ultimately, our simulation results provide interesting mechanistic insigths into structure-function relationships in the TLR4 signal transduction network, which enabled the identification of plausible rate limiting steps that most effectively control the performance of the system under diverse dynamical regimes.

### Information processing and biochemical reaction space of the signal transduction network

**Figure 1:**
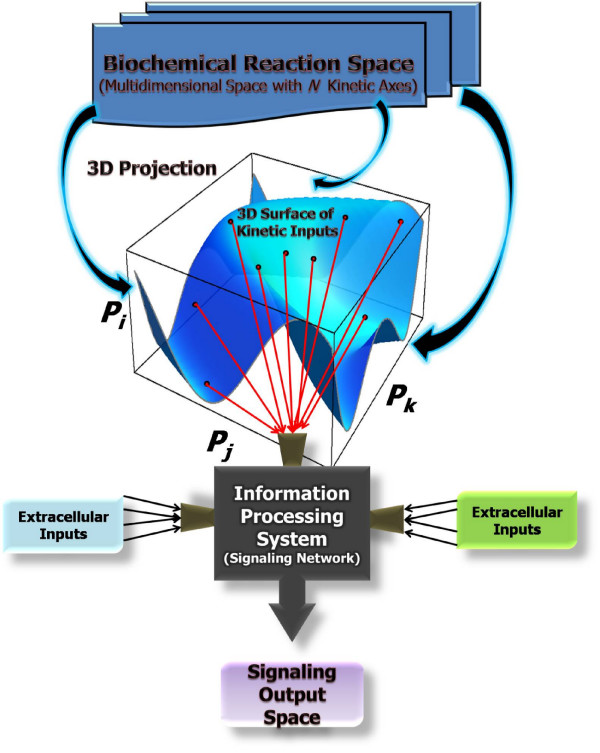
**Biochemical reaction space, and integrated information processing of inputs of diverse nature**. Signal transduction networks inhabit multidimensional biochemical reaction spaces encompassing repositories of kinetic information, which are integrated along with extracellular stimuli. Such heterogenous sources of information turn out to be simultaneously processed while being integrated, and a signaling ouput, which may determine a particular cellular state, must be robustly calculated according to the set of reaction rules and regulatory schemes encoded in the topology of the network. For simplicity purposes, in this schematic representation a 3D projection drawn from the multidimensional biochemical reaction space is illustrated. Each axis (*P*_*i*_, *P*_*j*_, *P*_*k*_) in this lower dimensional 3D space represents a reaction kinetic parameter (i.e. an enzyme catalitic rate), and collectively define a surface of inputs which are integrated with extracellulr stimuli, and processed in parallel by the signaling network, from which a given output is computed. Multiple points distributed across the 3D surface of kinetic inputs are sampled by the signaling network, which may represent distinctive reaction conditions stemming from thermal fluctuations in the cell environment, or mutational perturbations in the genetic encoding of the network. Ideally, however, several points distributed across a hypersurface embedded in the *N*-Dimensional reaction space are systematically sampled by a signal transduction network. In this study, while keeping a given extracellular stimuli constant, the biochemical reaction space is systematically explored around reference operative points via global and local perturbation strategies. In this way, an unbiassed statistical assessment of the robust properties and information processing capabilities of a canonical reaction network underlying TLR4 signaling events was performed.

To avoid any confusion or controversy regarding well stated systems biology concepts on cell signaling processes, it is important to make clear our notion of a signal transduction network as an information processing system, mainly because this may differ considerably from previous conceptualizations. Nevertheless, we believe our conceptualization provides a complementary view of the issue. For example, the notion of information processing applied in the context of intracellular signaling has traditionally been limited to the mechanistic explanation of how cellular behaviors are induced via the decodification, and subsequent intracellular propagation, of time variant/invariant physicochemical signals provided by extracellular stimuli (see for example [6,38-43]). Our intent here was to extend the scope of this notion, making it more suitable for systems-level robustness analysis of signal transduction networks. Our rationale focuses on the following arguments. *Given that the emergence of central cellular behaviors relies heavily on the robust performance of signal transduction networks, it follows that the information processing capabilities of these systems are primarily dependent on internal reaction parameters*. *In general, such parameters exhibit a natural tendency to behave like a set of random variables, resulting mainly from thermal fluctuations in the cell environment, and mutational perturbations in the genetic encoding of the system*. *Arguably, the internal reaction parameters of a signaling network stand for repositories of kinetic information that collectively define a biochemical reaction space inhabited by the system*. *Such a reaction space becomes an essential source of information carefully coupled to extrinsic stimuli that turn out to be processed according to the set of reaction rules encoded in the architecture of a signal transduction system, from which a proper cellular phenotype (i.e dynamic protein activation profiles and/or gene expression patterns) is calculated *(see Figure 1). Ideally, these should represent the basic tasks any information processing system, such as a signal transduction network, is expected to accomplish in a robust fashion. Under these considerations, it should be clear that we equate robust information processing capabilities of a signaling network with its capacity to reproduce particular (reference) dynamical trajectories of biochemical activity under random perturbations in its internal reaction parameters. Importantly, this is assessed here via standard metrics aimed at evaluating discrepancies between dynamical trajectories, and by means of rigorous statistical analysis (see the "Models and computational framework" section below). Our methodology can thus be seen as a coarse-grained strategy to assessing the information processing capabilitites of a complex reaction network, when monitoring the propagation of kinetic uncertainties throughout the system. This represents an alternative framework to that recently proposed methodology relying on Shannon's entropy (see [44]). Interestingly, that framework conceives a signaling network as a "communication channel", for which the associations between inputs and outputs result from a decomposition of their mutual information into different components.

## Methods

### Canonical reaction topology underlying TLR4-mediated signal transduction events

**Figure 2:**
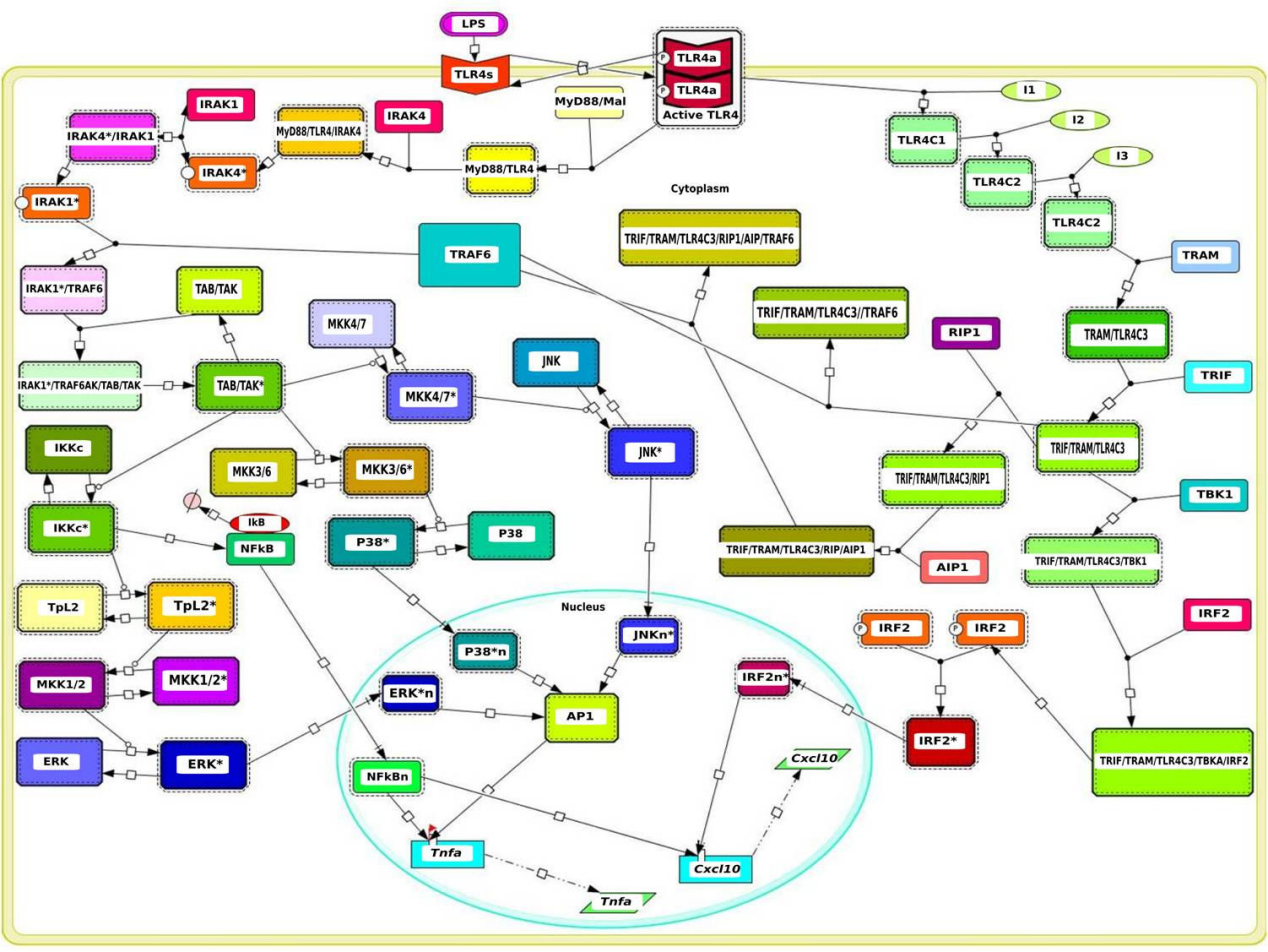
**Canonical reaction topology underlying TLR4-mediated signaling events**. This canonical topology was assembled according to well-documented studies on the reaction steps deployed during TLR4-mediated signaling in macrophages, in response to LPS stimulation. Our kinetic model accounts for the reaction dynamics of 76 molecular species, including single species and transiently-formed complexes resulting from the aggregation of two or more species. Some intermediate species are not illustrated; only key reaction components are shown. Our kinetic modeling approach is founded on basic principles of biochemical reaction, accounted for via simple mass action law (both first and second order kinetics) and generalizations of Michaelis-Menten reaction kinetics.

Within a rather short time window, LPS binding to TLR4 triggers two major intracellular signaling events rapidly propagated through the MYD88-dependent and TRAM-dependent reaction cascades, which display extensive crosstalking (see Figure 2). Activation of the MYD88-dependent cascade leads to induction of pro-inflammatory cytokines such as TNF*α *by means of JNK, p38, NF-*κ*B and ERK; whereas the TRAM-dependent cascade predominantly induces the expression of chemokines such as the IP-10 protein encoded in the *Cxcl10 *gene, via the interferon regulatory factor (IRF) [45]. A relatively limited number of existing dynamic modeling studies focus specifically on TLR4-mediated signal transduction. For example, pioneering simulation works have provided interesting mechanistic insights on diverse kinetic phenomena observed during temporal deployment of this signal transduction network, such as time delay responses [46], signaling flux redistribution [47], and preconditioning behavior [48,49]. Based upon the information provided by these theoretical studies and the data reported in recent review articles about key architectural features of this signaling network (see for example [31-37]), we assembled a well-informed mathematical representation of the complex web of biochemical reactions that are likely to sustain the information processing capabilities of this signal transduction system. Our modeling framework is grounded on ordinary differential equations incorporating first and second order reactions for representing intracellular signaling fluxes, as well as Hill-like saturation kinetics accounting for highly non-linear reaction schemes taking place at the level of ligand-receptor interactions and transcriptional activation (see "Models and computational framework" section below, and Additional file 1 for a detailed description of the mathematical structure of the network model). The total number of reaction species modeled amounts to 76, including a TLR4 in both a susceptible and an activated form, MYD88 and TRAM adapters along with their associated molecules, hypothetical intermediates upstream to TRAM which have been inferred computationally in [46,47], intermediate and effector kinases (i.e. MKK4/7, JNK, MKK3/6, p38, TpL2, MKK1/2, ERK), the associated and dissociated forms of NF-*κ*B and I*κ*B, and two important mRNAs transcribed from the *Tnfα *and *Cxcl10 *pro-inflammatory genes (see Figure 2). We also assumed a time variant concentration of LPS following an exponential decay profile as an alternative hypothesis to that simulated intrinsically stable dynamic regime of LPS proposed in a recent study of TLR4 activation kinetics ([48]). Nuclear export and import dynamics from the cytoplasm of some reaction species were modeled via simple first order kinetics, hence, volume-dependent scaled coefficients of transport were neglected for simplicity purposes. Moreover, within the narrow time window simulated, our modeling framework assumes that simple first order reaction kinetics govern dephosphorylation processes. In this way, dephosphorylation of a substrate was only dependent on its own concentration and the dephosphorylation rate. Furthermore, we lumped together into single reaction steps multisite phosphorylation processes, which might not represent key rate limiting steps in the cascades included in our model. We therefore have equated multisite phosphorylation steps with full kinase activation, which might constitute a truly rate limiting step during signal processing. It is also worth saying that an explicit mathematical representation of the dynamics of ATP was not considered; instead, we assumed it to be in a steady state. This is standard practice in kinetic modeling and is implemented for simplicity purposes. Our mathematical representation of the whole reaction scheme defines a multidimensional biochemical reaction space encompassing 116 kinetic coefficients (axes), including transition rates between receptor states (susceptible ⇌ activated), production and degradation rates of receptors, association/dissociation rates among intracelular molecular species, phosphorylation/dephosphorylation rates, nuclear import/export rates, maximal transcriptional rates, transcriptional efficiencies, Michaeles-Menten constants, cooperative coefficients, and mRNA degradation rates. Reaction kinetic values for this signaling system have so far proven extremely difficult to assess under well controlled experimental conditions. Therefore, our massive amounts of computationally predicted values of internal reaction parameters for this signaling network might provide a glimpse on the kinetics of the system under different cellular states. Moreover, despite obvious simplifying assumptions about the intricacies of the reaction steps involved, our mathematical representation captures core design principles of the signal transduction network. This is because our model was validated with dynamic experimental data (time courses) from wild-type target transcriptional readouts, which have been shown to be actively modulated, in quantitative terms, by the reaction cascades accounted for in our proposed scheme (see below). Critically, our simulated time window was limited to an interval spanning 120 minutes, a time scale during which critical transient transcriptional readouts are realized as a result of rapidly mounted innate immune responses ([47]). Furthermore, the transient features exhibited by the network during such time period emerge primarily as a consequence of intrinsic processes guided by the intracellular regulation of TLR4 signaling in response to LPS. This is opposed to those extrinsic processes triggered by autocrine and paracrine stimuli provided by anti-inflammatory cytokines (i.e. IL-10 and TGF-beta), which entails the temporal deployment of complex regulatory schemes such as (+/-) feedback control. Presumably, within the narrow temporal window of TLR4 activation in response to LPS stimulation, which is the focus of our modeling framework, the initial signaling phase might not be heavily dependent on the complex feedback dynamics that are subsequently displayed by the NF-*κ*B regulatory module [50]. Such dynamics, instead, should play a major role in reliable control of a delayed (secondary) signaling phase in response to LPS stimulation (see for example [51]). Interestingly, the presence of two signaling phases in this crucial immune cellular process might represent very distinct episodes of signaling fluxes, carrying particular information, that differentially modulate in quantitative terms the transcriptional readout of specific gene batteries.

## Results

### General robustness properties of the signal transduction network in different regions of the biochemical reaction space

**Figure 3:**
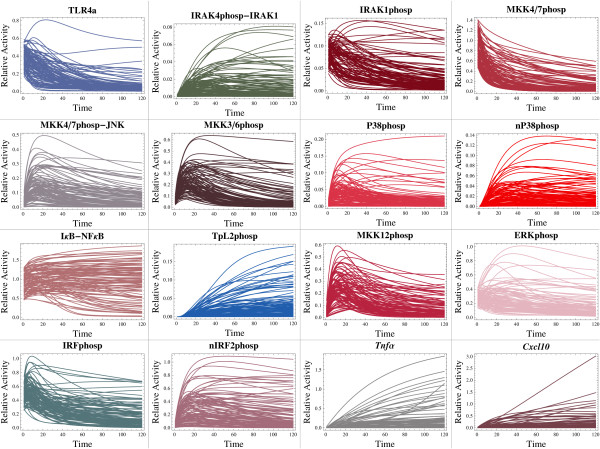
**Ensemble of hypothetical dynamical trajectories**. A wide spectrum of hypothetical but biologically plausible dynamical trajectories accessible to the reaction network was simulated. An ensemble encompassing 100 different trajectories accessible in widely scattered regions of biochemical reaction space were propagated from very particular initial conditions. The figure illustrates a subset of individual dynamical trajectories displayed by some key reaction species modeled (100 trajectories for each species are shown). Most of these simulated trajectories were found to be capable of displaying transient or sustained dynamical features, which have been reported to be typical dynamical behaviors emerging during crucial intracellular signaling events.

**Figure 4:**
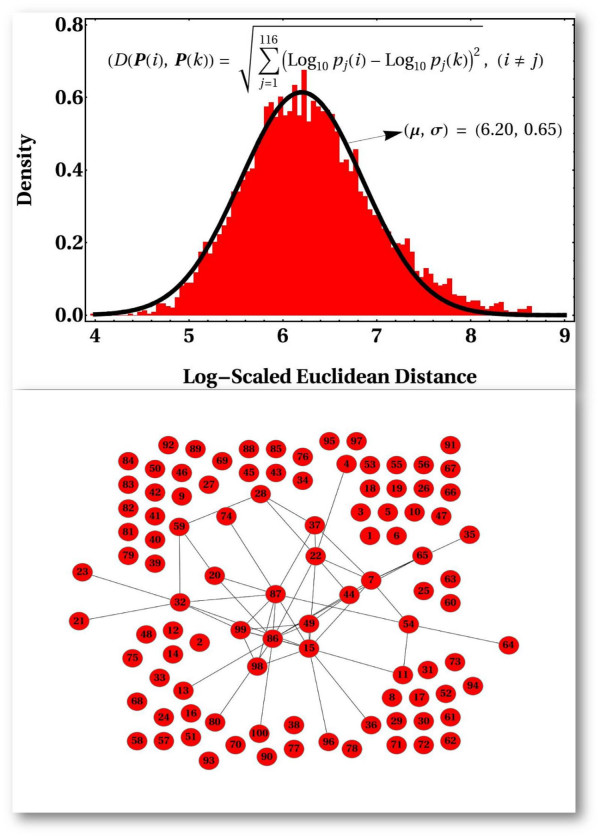
**Metric relations among reference dynamical trajectories distributed in different regions of biochemical reaction space**. To more clearly appreaciate the possible metric relations among the 100 parameter configurations (reference points) distributed throughout biochemical reaction space that were selected, we calculated all possible distances (via the metric shown in the top panel) among configurations. We then fit the empirical distribution to a theoretical Normal distribution with parameters *μ *= 6.20 and *σ *= 0.65. With this information at hand, we constructed the graph shown in the bottom panel. This graph provides an interesting graphical notion of the possible metric relations among configurations in parameter space. We implemented a decision rule in order to construct the input adjacency matrix (a binary matrix) of the graph: if any element of the matrix *A*, *a*_*ij*_, containing Log-scale Euclidean distances (see metric in top right panel) among parameter configurations is *a*_*ij *_⋜ *μ *- 2 * *σ *then *a*_*ij *_→ 1; otherwise *a*_*ij *_→ 0. The calculated graph is meant to illustrate how likely one point in parameter space (here represented by a node in the graph) can be accessed from another one via multiple perturbations. For example, pairs of linked nodes indicate that such configurations are relatively close in biochemical reaction space, and thus, one configuration might be accessed from the other via, perhaps, few random changes. In top right panel, *P*(*i*) and *P*(*k*) stand for any parameter configuration *i *or *j *included in the ensemble of trajectories analyzed.

**Figure 5:**
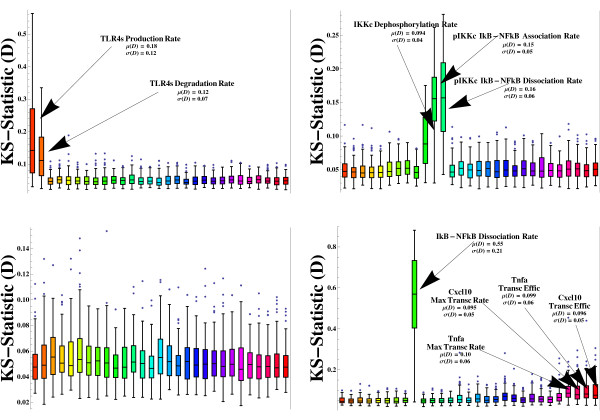
**Spectrum of global sensitivities**. D values calculated via our MPSA scheme, described in the Methods section, are shown, which provide a detailed idea on the sensitivity of the reaction network to variation in particular parameters, when the remaining parameters were varied simultaneously. Bar plots are shown for each reaction parameter modeled, summarizing the statistical tendency of the D-values calculated for each parameter. 116 bars are shown, each associated to a given reaction parameter.

Our first round of numerical experiments was designed with the main goal of exploring the intrinsic robustness properties of the whole integrated reaction network. We computationally reconstructed a rather limited ensemble of 100 different signaling regimes or dynamical trajectories (i.e. the set of 76 individual temporal profiles for the reaction species modeled, which is associated with a given point in parameter space) attainable by the network (see Figure 3). We randomly explored the parameter space looking for solutions in which some reaction species undergoing, for example, covalent modifications (i.e. phospho/dephosphorylation) displayed particular dynamic features similar to previously simulated, and experimentally reported, signaling outputs. Specifically, we focused on trajectories displaying biologically plausible dynamical signatures, such as sustained and transient dynamics of molecular activity with identifiable signaling peaks in some cases. Our simulated reference trajectories were thus required to match, at least qualitatively, distinct signaling outputs previously reconstructed computationally from experimental data (see for example [14,15,28]). Under these considerations, such an ensemble of reference trajectories can be thought of as being congruent with a plausible spectrum of cellular states attainable by, for example, a macrophage, which may be a natural operative condition (i.e. phenotypic plasticity) of many types of immune cell lineages ([52]). Alternatively, such an ensemble of dynamical trajectories can be seen as a set of widely scattered points in the multidimensional biochemical reaction space (see Figure 4), with some points being closely related and defining small neighborhoods in biochemical reaction space. As noted above, we randomly explored the parameter space according to a previously defined range of variation assigned to each reaction parameter (see Additional file 1 for a detailed description of parameter ranges); ranges of variation were constrained based on previous simulation results obtained from random scrutiny of the parameter space (personal observations, data not shown), and biological intuition. Moreover, each reference dynamical trajectory was propagated from a particular set of intital conditions (see Additional file 1 for a detailed description), which were also constrained based on previous simulation results (personal observations, data not shown) and biological intuition. Initially, thousands of simulated trajectories were carefully monitored both manually and systematically in order to assemble our final ensemble of biologically plausible dynamical trajectories. Comprehensive statitistical analyses were performed over our limited ensemble of reference trajectories. Ultimately, by following this computational methodology, we were able to conduct a series of well controlled *in silico *experiments that allowed us to probe the intrinsic robustness properties of the network, under different hypothetical scenarios of biochemical activity. We implemented a global (non-orthogonal) perturbation scheme, also known as multiparametric sensitivity analysis (MPSA) (see the "Models and computational framework" section below). This computational methodology provides the means for conducting efficiently systematic rounds of perturbations in each of the 100 reference points (reference parameter configurations) distributed throughout parameter space. Each reference parameter configuration was subjected to a round of 5000 simulataneous perturbations; that is to say, 5000 newly assembled parameter configurations surrounding each reference point in parameter space were generated. To do this, we first performed uncertainty analysis consisting of Monte Carlo simulations based on the efficient Latin Hypercube Sampling (LHS) scheme, followed by sensitivity analysis, which allowed the identification of those reaction parameters most critically involved in the global performance of the reaction network (see [23,53,54], and the "Models and computational framework" section below). Importantly, under this framework the robust information processing capabilities of our model reaction network were properly evaluated by means of a detailed statistical analysis of the system's global sentivities. We analyzed the distributions of the D statistics calculated from Kolmogorov-Smirnov (KS) tests performed by means of the afortmentioned perturbation approach. Briefly, a KS test is intended to evaluate the global sensitivity of the system's output with respect to perturbations targeting individual parameters. This test specifically provides the means for evaluating the cumulative frequency of the observations (parameter values) as a function of class, and for calculating the maximum vertical distance between cumulative frequency distribution curves for *m *acceptable and *n *unacceptable cases of any given parameter *θ*_*j *_(see the "Models and computational framework" section below). Figure 5 illustrates a series of box plots summarizing the overall statistical tendency of the D values calculated for each reaction parameter of the network model, over the ensemble of 100 dynamical trajectories that were systematically perturbed. Here, it is worth noting that for each perturbation study, the perturbed signaling trajectories were compared only with a corresponding reference trajectory; being such a trajectory a member of the ensemble of 100 trajectories analyzed. In general, our analysis indicates that the network is capable of reproducing reference dynamical trajectories of biochemical activity relatively well when their associated points in parameter space are systematically perturbed. This can be inferred by observing the excess of small average D-values associated to each reaction parameter. Interestingly, a notable statistical tendency with respect to the system's dynamical behavior was revealed. For example, the signaling network was found to be moderately and extremely sensitive to random perturbations in few reaction parameters. For instance, the parameters related to the *Dephosphorylation Rate of the IKK-complex*, and the *Maximal Transcriptional rates *and *Transcriptional Efficiencies *associated to the *Tnfα *and *Cxcl10 *genes can be categorized as moderately sensitive parameters, with average D-values ranging between 0.09 and 0.11. On the extreme side of the sensitivity spectrum, we found that the parameters related to the *Production *and *Degradation rates *of the *TLR4 Susceptible Form*, the *Association *and *Dissociation Rates between Phosphorylated IKK-complex and IkB-NFkB*, and the *Dissociation Rate between IkB and NFkB*, represent extremely critical (sensitive) points of the proposed reaction mechanism, with average D-values ranging between 0.12 and 0.58. Furthermore, our statistical analysis also revealed that the variability of the D-values for those parameters categorized as moderately and extremely sensitive were found to be extremely large, as indicated by both the height of bars and their corresponding whiskers. This result strongly suggests that the robustness properties of the network can be highly variable depending on its current position in biochemical reaction space. It is also interesting to analyze our simulation results from the viewpoint of sloppy and stiff multidimensional parameter spaces [11,12]. According to this well-supported theoretical framework, we may conclude that our proposed reaction scheme functions as a highly sloppy information processing system capable of performing robustly, despite undergoing simultaneous random perturbations in its internal reaction parameters. However, some stiff axes were found to be a defining feature of this multidimensional space, along which random perturbations lead predominantly to dramatic changes in the global dynamical behavior of the system. Therefore, such stiff axes in biochemical reaction space constitute key variational constraints of the proposed reaction mechanism. Following this direction, our simulation results strongly suggest that those biochemical processes relying on the reaction parameters identified as critical points of the network, should represent the rate limiting steps that most effectively control the global dynamical behavior of the system. We thus predict that such critical reaction steps represent ideal candidates for manipulating the dynamic activity of the TLR4 signaling network via multi-target therapeutic strategies, which might provide the means for modulating quantitatively innate immune cellular responses in an efficient manner.

#### Variability of key individual dynamical trajectories

**Figure 6:**
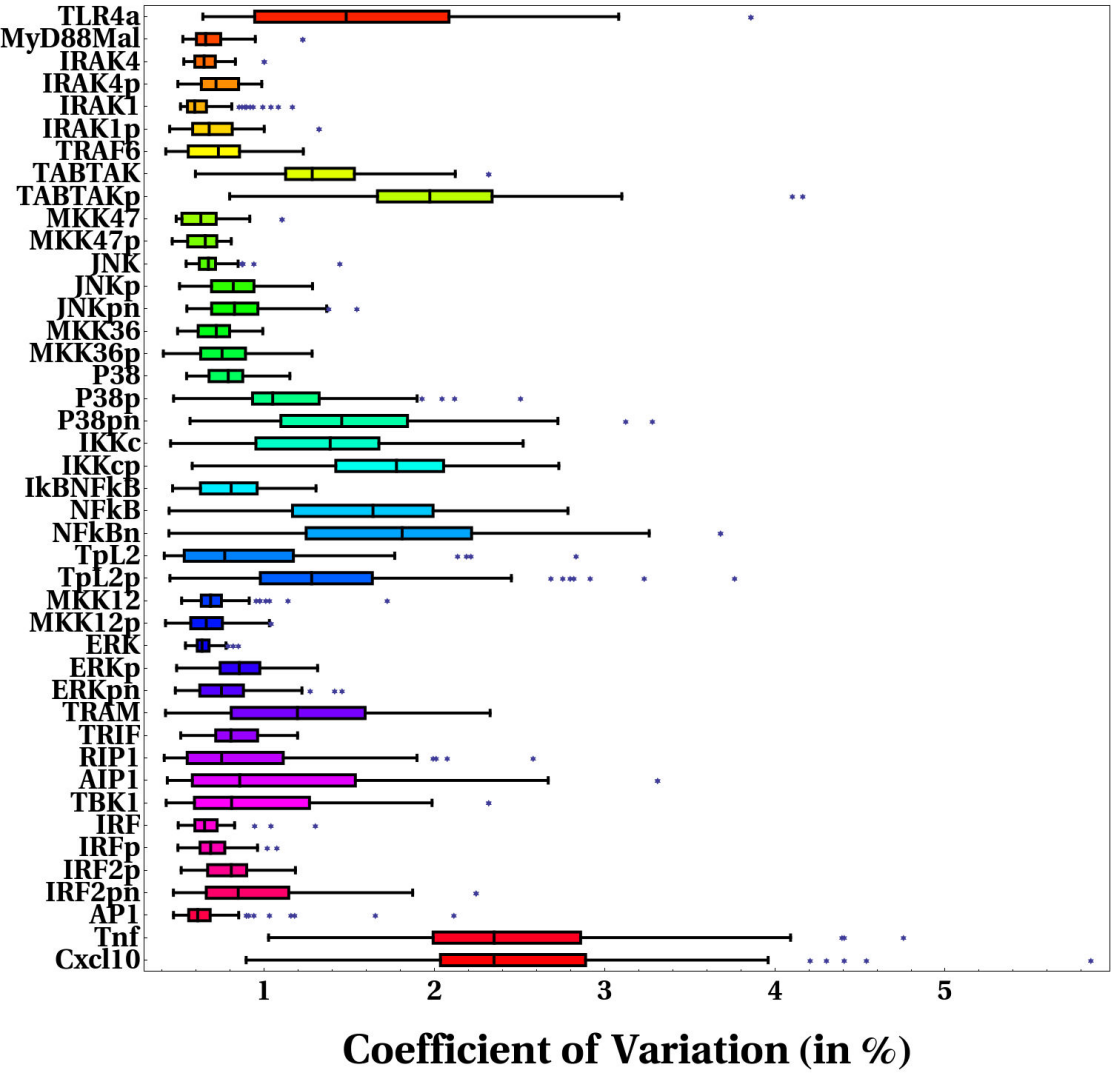
**Spectrum of variabilities for individual dynamical trajectories of each reaction species modeled**. Coefficient of variation were calculated for the discrepancies, from reference trajectories, of individual trajectories displayed by each reaction species modeled in the face of global perturbations. Results from only a subset of key reaction species are shown. The results shown correspond to those configurations that were found to be robust to random, simulataneous perturbations.

Further statistical analyses were performed to characterize the variability of the dynamical trajectory displayed by each individual reaction species modeled, upon systematic perturbation of the entire biochemical reaction space. We calculated the coefficient of variation of the discrepancies of individual trajectories from the corresponding reference trajectory. A simple Euclidean metric was implemented to evaluate such discrepancies (see the "Models and computational framework" section below); again, this was carried out for each reference trajectory included in the final ensemble of 100 trajectories simulated. In this analysis we focused on those dynamical trajectories categorized as robust/insensitive according to our previous MPSA. This analysis provides primary information on key variational constraints in the network's dynamical behavior. Figure 6 illustrates the results of our statistical analysis, wherein a highly heterogenous spectrum of variability can be readily appreciated, indicating that not all the dynamical trajectories of individual reaction species tend to vary similarly upon global perturbation of the biochemical reaction space. Notably, the temporal trajectory of some reaction species was found to be more variable than others. Note for example that the most upstream (i.e. TLR4 activated form) and the most downstream reaction species (*Tnfα *and *Cxcl10*) along the signaling cascades modeled, exhibited a remarkable tendecy to vary upon global perturbations. This can be explained by noting that the reaction rules underlying ligand-receptor and transcriptional kinetics involve highly non-linear processes related to cooperativity interactions and saturation phenomena. Therefore, it should be expected for multiple combination of perturbations in the kinetic parameters driving such non/linear reaction processes to exert drastic changes in the dynamical trajectories of the system. Alternatively, we also found that some intermediate reaction species along the signaling cascades analyzed exhibit a considerable tendency to vary; although some notable differences were observed. For example, a large number of species associated to the MyD88-dependent signaling pathway, namely, TABTAK, TABTAKp, P38pn, IKKc, IKKcp, NF*κ*B, NF*κ*Bn, TpL2p, were found to vary considerably; whereas only the reaction species TRAM, in the alternative reaction cascade downstream the TLR4, was found to vary significantly. It is tempting to speculate on these results based on the fact that a larger density of coupled biochemical reactions along the MyD88-dependent signaling pathway occurs during the time scale considered in our simulations. From this, it then follows that a stronger dynamical coupling of biochemical reactions (functional dependencies/linkages) through this pathway might lead to considerably larger effects when multiple perturbations are propagated dynamically.

#### Comparison of total parameter variation spectra

**Figure 7:**
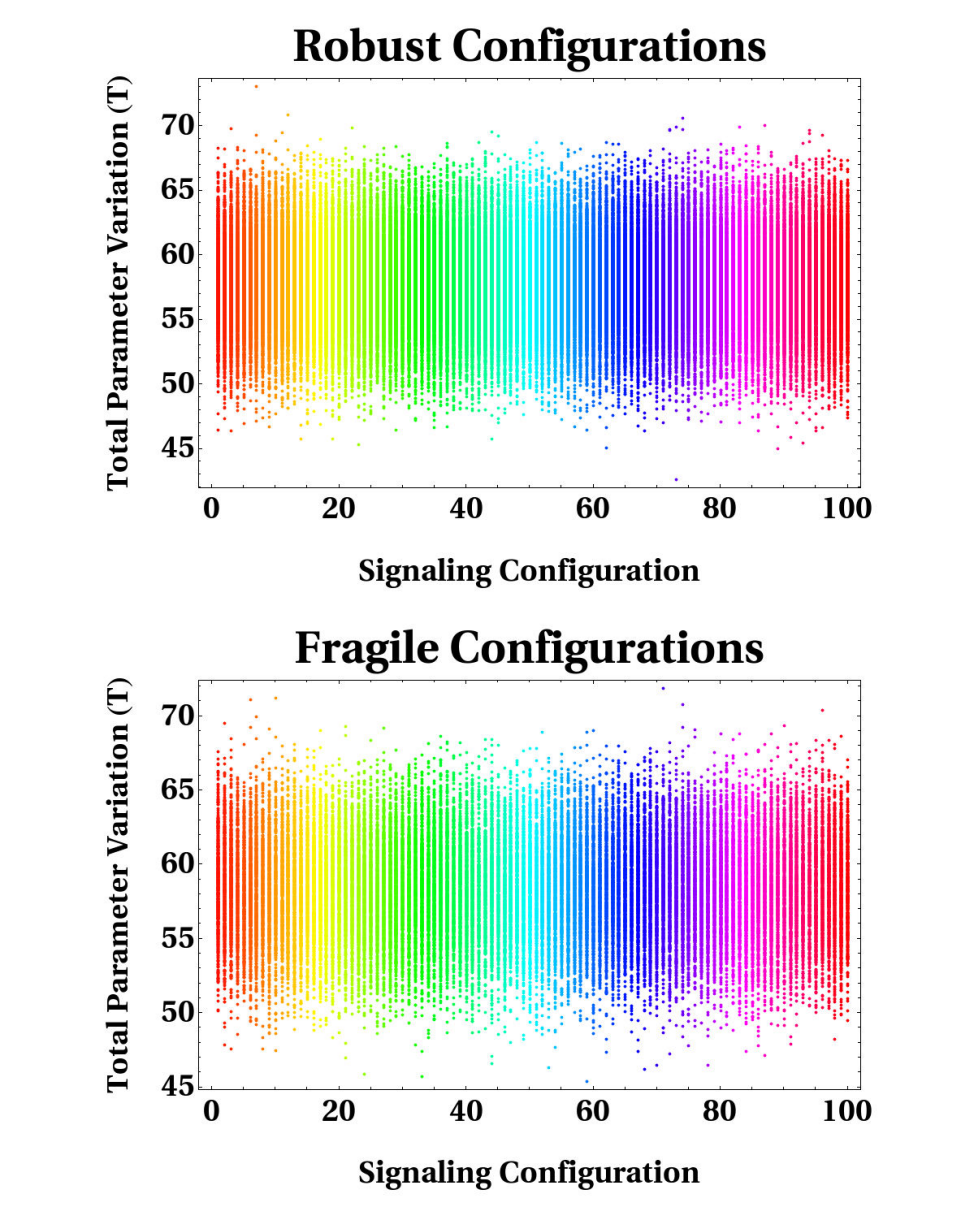
**Spectra of total parameter variation**. Total parameter variation (T) represents a measure providing a quantitative notion of the order of magnitude in the variation of a perturbed parameter configuration obtained from a reference one. Two spectra of T values are illustrated, which were assembled for both robust and fragile configurations. Each line of vertical points indicates the distribution of T values calculated when a reference point in parameter space was subject of global perturbations. Note that each spectrum is composed of 100 distributions of T values.

Finally, we sought to quantify the capacity of the network of absorbing large fluctuations in internal reaction parameters, and in different regions of biochemical reaction space. We assessed and compared the spectra of total parameter variation (T) for those configurations that were identified as robust and fragile (sensitive) according to our previous MPSA. This measure provides a quantitative notion of the order of magnitude in the variation of a perturbed parameter configuration obtained from a reference one (see the "Models and computational framework" section below). The analysis was performed in each of the 100 dynamical trajectories previously assembled. In Figure 7, every vertical line of points illustrated in each panel stands for a distribution of T values calculated for each reference dynamical trajectory defined by a given point in parameter space. To test for statistical differences between the two spectra shown in Figure 7, we ran Mann-Whitney tests between robust and fragile distributions. Of the 100 statistical tests performed, we found that 67% of them yielded p-values < 0.05, thus indicating that, in general, the two spectra tend to differ significantly. However, a simple graphical comparison between the two spectra indicates that a similar global tendency appears to exist (see ranges of variation, for example). In other words, this seems to suggest that the capacity of the signal transduction network of absorbing random perturbations in the whole set of internal reaction parameters may be quite similar under both robust and fragile conditions. At first glance, this observation appears counterintuitive, because it would be expected that for those perturbed configurations categorized as robust/insensitive, small quantitative departures from the reference parameter configuration should be a prevailing statistical regularity. This observation is consistent with the idea that the robust dynamical performance of the network should be more heavily dependent on the direction towards which random perturbations are induced in the biochemical reaction space, than on the magnitude of the perturbation itself.

### Robustness of particular input-output maps: effects of local and global perturbations at the level of individual transcriptional outputs

Upon extensive exploration and statistical characterization of general robustness properties inferred from hypothetical, but biologically plausible dynamical trajectories displayed by the network, we then focused on a detailed analysis of particular input-output maps embedded in the model reaction scheme.

**Figure 8:**
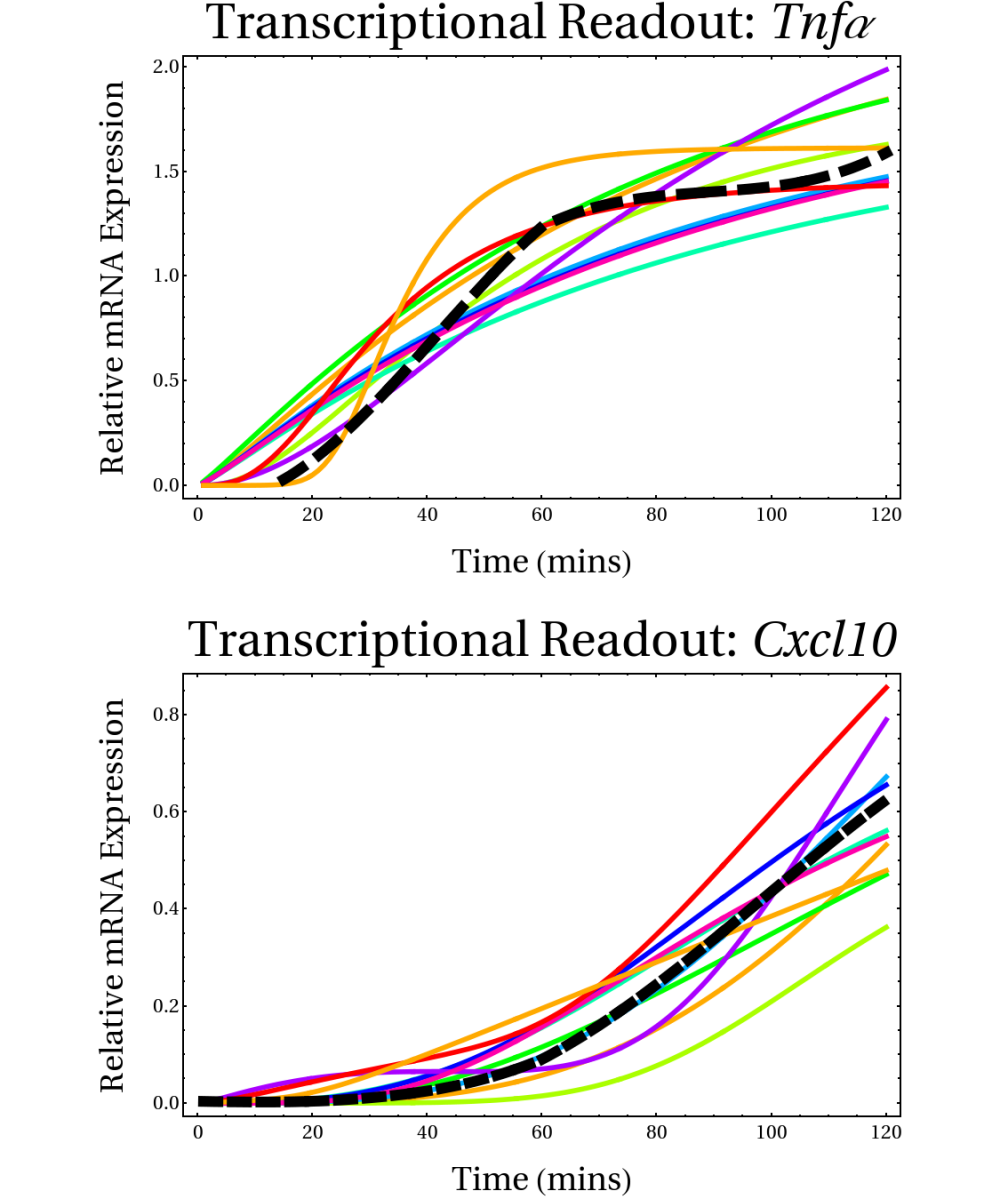
**Experimentally reported and simulated transcriptional readouts**. Black dashed trajectories indicate experimentally reported transcriptional activation profiles during a short time window of 120 minutes. Color-coded trajectories stand for simulated trajectories obtained from an extensive exploration of biochemical reaction space by means of Monte Carlo simulations and a pseudo-random search algorithm. Experimental data encompassed only 6 time points that were sampled in cell cultures during the time window of 120 minutes. We performed non-linear interpolation in order to infer the relative expression levels of each gene every minute during the time window. This strategy allowed us to further constrain our simulations.

Specifically, we sought to characterize the robustness of the temporal trajectory of the two transcriptional readouts incorporated in the signaling network model. *Tnfα *and *Cxcl10 *represent crucial outputs required for the appropriate development of pro-inflammatory responses, which are critically modulated by the upstream reaction cascades activated upon LPS stimulation. Figure 8 shows the temporal profile for the transcriptional activation of these genes. It is worth emphasizing that our modeling framework only accounts for transcriptional activation processes during the short time window simulated. Accordingly, it was assumed that transcriptional activation was mainly driven by the activity of ERK, P38, NF*κ*B, JNK, and IRF. Consequently, transcriptional repression effects were deliberately neglected. Before conducting the perturbation experiments, several random explorations of the biochemical reaction space were first performed to identify different parameter configurations capable of reproducing the reported experimental data (Figure 8, black-dashed trajectories). Monte Carlo simulations were thus performed taking as reference the set of kinetic data (both initial conditions and parameter ranges) used for simulating the ensemble of reference dynamical trajectories previously constructed (see Additional file 1). Moreover, based on this same reference ensemble of kinetic data, random searches through parameter space via a pseudo-random search algorithm (PRSA) were also performed, as described in [55]. This time, we constructed a small ensemble of 10 reference parameter configurations widely scattered in biochemical reaction space (see Additional file); each reference parameter configuration was able to reproduce relatively well, in quantitative terms, the empirical data (Figure 8, color-coded trajectories). We focused on this ensemble for conducting local and global perturbation analysis with respect to the transcriptional activation profiles of the *Tnfα *and *Cxcl10 *pro-inflammatory genes.

#### Local perturbation analysis of transcriptional outputs

The computational strategy designed for systematically exploring orthogonal (i.e. local) perturbations in each of the 10 reference points previously sampled is described below (see the "Models and computational framework" section below). In this analysis, perturbations were restricted to those reaction parameters sustaining only signaling fluxes, while transcriptional parameters were maintained unperturbed. In this way, we were able to analyze the quantitative effects at the level of transcriptional readouts of small perturbations impinging upon single reaction kinetics of the upstream signaling cascades. Tables 1 and 2 summarize the calculated overall state sensitivity coefficients for a range of magnitudes of the perturbations induced (∀ Δ *P *∈ {0.1, 0.2, 0.3, 0.4, 0.5}, see the "Models and computational framework" section below) in each single reaction parameter. Importantly, the calculated coefficients were found to be remarkably small in comparison to other calculated values reported in a previous simulation study of the MAPK signaling module (see [55]). This observation clearly indicates that the reaction mechanism underlying TLR4-mediated signaling behaves as a robust information processing system in the face of small quantitative fluctuations in individual reaction parameters. In other words, this inherent robust condition endows the signaling network, under very particular mutational conditions (i.e point mutations) and within a rather short time window, with the capacity of converting an external stimuli into highly reproducible transcriptional readouts. We note that the two transcriptional readouts considered tend to exhibit the same pattern of sensitivity to most reaction parameters. Interestingly, however, some parameters were found to be more determinant for one transcriptional readout than for the other. For example, the temporal expression profile of *Tnfa *was found to be relatively more sensitive to variations in *k*31*f *(*Association Rate between TLR4I1 and I2*), *k*35*r *(*Dissociation Rate of the Complex TLR4I1I2I3TT from RIP*), *ksa *(*Transition Rate from Susceptible to Active TLR4*), and *k*14*r *(*Dissociation Rate of the Complex TABTAKp - MKK3/6*); whereas the dynamical trajectory of the transcriptional readout of *Cxcl10 *exhibited a more pronounced sensitivity to perturbations in *k*40*f *(*Association Rate of TLR4I1I2I3TTTTBK1 with IR*F), *k*42*f *(*Dimerization Rate of IRFp*), *kas *(*Transition Rate from Active to Susceptible TLR4*), and *k*1*r *(*Dissociation Rate of MYD88/Mal - TLR4*). Although informative, results from local (orthogonal) perturbation analysis provide only limited insight on the variational constraints and systems-level properties of the signal transduction network. We next performed a global perturbation analysis to this aim, as described below.

**Table 1:** Statistics on overall state senstivities from local perturbation experiments for the transcriptional output *Tnfα*. Values shown were averaged over the ensemble of 10 reference parameter configurations evaluated. Mean-D (mean D Statistic); SD-D (standard deviation of D Statistic)

Parameter	Δ*P *= 10%	Δ*P *= 20%	Δ*P *= 30%	Δ*P *= 40%	Δ*P *= 50%	Mean-D	SD-D
*Kb*	0.003369	0.002241	0.001876	0.001177	0.000868	0.001906	0.002154
*n*	0.001355	0.0007648	0.0006529	0.001581	0.000949	0.001060	0.001100
*k*21*cat*	0.001128	0.001713	0.000659	0.000536	0.000595	0.000926	0.001031
*k*22*f*	0.001314	0.001137	0.000969	0.000379	0.000388	0.000837	0.001662
*k*16*r*	0.001292	0.001043	0.000711	0.000502	0.000401	0.000790	0.001557
*k*23*cat*	0.001019	0.001376	0.000464	0.000386	0.000461	0.000741	0.001361
*kps*	0.001065	0.000997	0.000536	0.000436	0.000414	0.000690	0.001315
*k*33*r*	0.001334	0.000744	0.000484	0.000487	0.000373	0.000685	0.001388
*k*23*r*	0.000434	0.001110	0.000619	0.000768	0.000299	0.000646	0.000998
*k*19*r*	0.001099	0.000706	0.000749	0.000332	0.000288	0.000635	0.001372
*k*7*r*	0.001191	0.000658	0.000469	0.000422	0.000324	0.000613	0.001395
*k*23*f*	0.001247	0.000569	0.000588	0.000358	0.000284	0.000609	0.001382
*k*18*r*	0.000668	0.000753	0.000676	0.000545	0.000345	0.000597	0.000918
*k*31*f*	0.000721	0.000701	0.000571	0.000461	0.000453	0.000581	0.001235
*k*32*f*	0.000480	0.000857	0.000546	0.000561	0.000429	0.000574	0.000904
*k*24	0.001064	0.000625	0.000520	0.000304	0.000266	0.000556	0.001329
*k*35*r*	0.001483	0.001000	0.000117	0.000105	0.0000739	0.000556	0.001630
*ksa*	0.000919	0.000725	0.000464	0.000314	0.000251	0.000535	0.001119
*k*8	0.000638	0.000616	0.000533	0.000559	0.000183	0.000506	0.000873
*k*14*r*	0.001026	0.000601	0.000251	0.000245	0.000340	0.000493	0.000748

**Table 2:** Statistics on Overall State Senstivities from Local Perturbations Experiments for the Transcriptional Output *Cxcl10*. Values shown were averaged over the ensemble of 10 reference parameter configurations evaluated. Mean-D (mean D Statistic); SD-D (standard deviation of D Statistic)

Parameter	Δ*P *= 10%	Δ*P *= 20%	Δ*P *= 30%	Δ*P *= 40%	Δ*P *= 50%	Mean-D	SD-D
*Kb*	0.007726	0.005217	0.006069	0.004233	0.004070	0.005463	0.007836
*n*	0.005616	0.004478	0.003862	0.003824	0.003714	0.004299	0.007452
*k*40*f*	0.002317	0.002146	0.002607	0.002518	0.002238	0.002365	0.006197
*k*42*f*	0.003415	0.002004	0.001858	0.001902	0.001834	0.002203	0.004786
*k*21*cat*	0.002860	0.002644	0.001749	0.001634	0.001206	0.002019	0.002098
*k*16*r*	0.004237	0.002285	0.001129	0.000868	0.000731	0.001850	0.002971
*kps*	0.003663	0.002090	0.001305	0.001196	0.000839	0.001819	0.002631
*k*23*cat*	0.003425	0.002015	0.001266	0.001124	0.000651	0.001696	0.002657
*k*33*r*	0.003194	0.002201	0.001196	0.001030	0.000760	0.001676	0.002669
*k*23*f*	0.003989	0.002014	0.001034	0.000723	0.000590	0.001670	0.002982
*k*19*r*	0.002917	0.001721	0.001572	0.000947	0.000739	0.001579	0.002649
*k*8	0.002686	0.001737	0.001478	0.000968	0.000540	0.001482	0.002459
*k*22*f*	0.002733	0.001615	0.000998	0.001147	0.000826	0.001464	0.002661
*k*23*r*	0.002807	0.001821	0.001181	0.000786	0.000697	0.001458	0.002383
*k*7*r*	0.002639	0.001675	0.001317	0.000935	0.000648	0.001443	0.002659
*k*32*f*	0.001537	0.002349	0.001265	0.001116	0.000824	0.001418	0.001876
*k*24	0.002710	0.001463	0.001237	0.000729	0.000597	0.001347	0.002629
*kas*	0.001528	0.002123	0.001111	0.000830	0.000810	0.001280	0.002070
*k*1*r*	0.001324	0.001825	0.001397	0.000960	0.000753	0.001252	0.001656
*k*18*r*	0.001279	0.001625	0.001444	0.000881	0.000708	0.001187	0.001692

#### Revealing the global perturbation landscapes of transcriptional outputs

**Figure 9:**
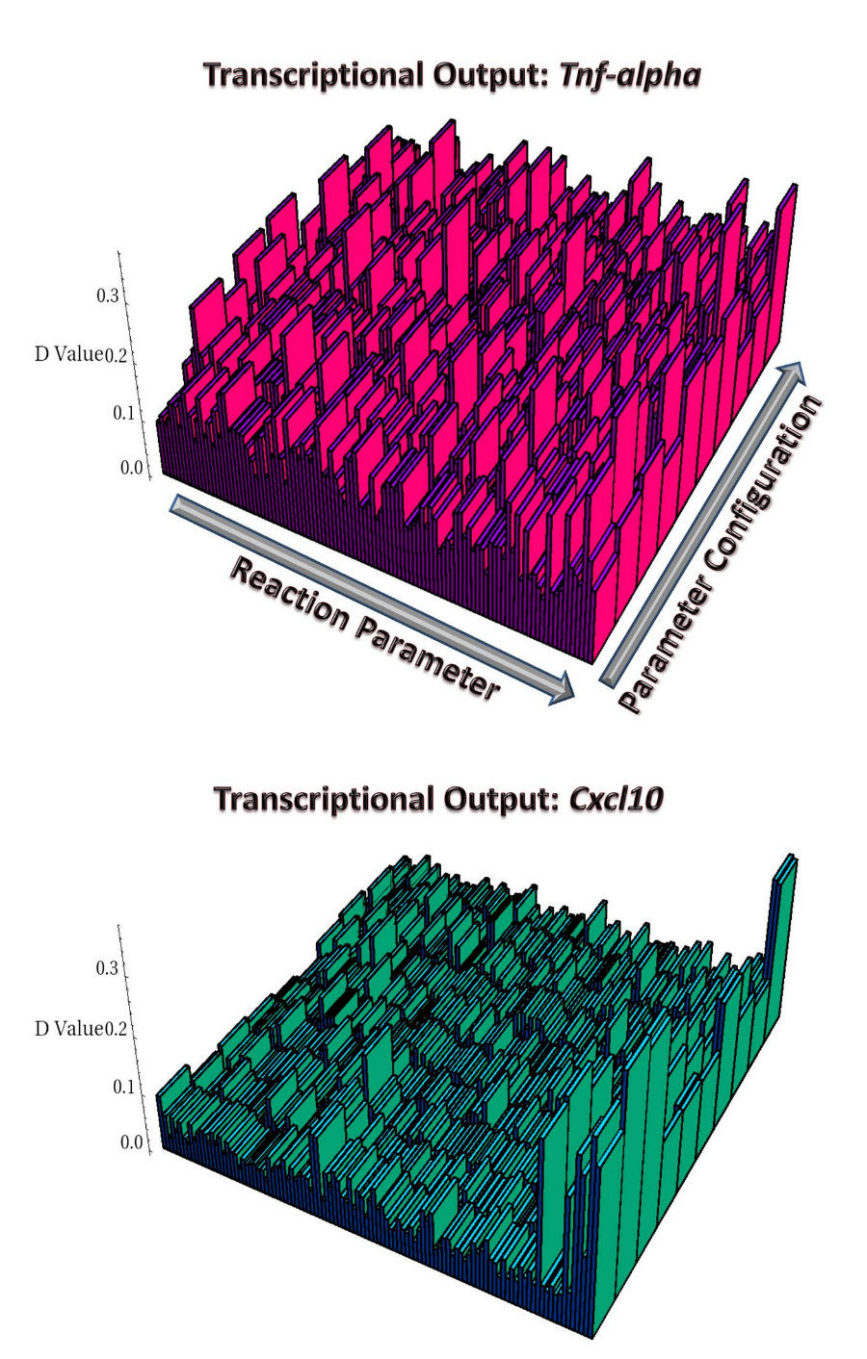
**Global perturbation landscapes of transcriptional readouts**. D-values calculated for each internal reaction parameter modeled, in ten different regions of biochemical reaction space. Each landscape is composed of 3 axes. The reaction parameter axis accounts for each reaction parameter directly or indirectly influencing the transcriptional activation of one of the pro-inflammatory genes. In this way, such axis involves 108 reaction parameters, rather than the whole set of model parameters which amounts to 116. The parameter configuration axis encompasses the 10 points in parameter space analyzed that were found to reproduce relatively well the reported transcriptional outputs. The Z axis indicate the magnitude of the global sensitivities, which are given by the D-values calculated from our global perturbation analysis.

In this analysis, we considered global (non-orthogonal) perturbations systematically induced on each of the 10 reference points distributed in biochemical reaction space. We generated 5000 perturbed configurations from each reference point, following our global perturbation strategy. This set of perturbation experiments were carried out to characterize the global perturbation landscapes based on well identified input-output relationships. We thus calculated a large number of D-statistics (obtained via the MPSA approach) for each simulated transcriptional output. That is, D-statistics were computed for each parameter in 10 different regions of the biochemical reaction space. Briefly, the perturbation landscapes illustrated in Figure 9 provide systems-level insights into the robust properties and information processing capabilities of the network, but this time in terms of particular input-ouput maps embedded in the reaction system. In these landscapes the reaction parameter axis, which ranges between 1-108, indicates the set of model parameters influencing directly or indirectly the transcriptional activation of each gene; whereas the parameter configuration axis, ranging between 1-10, describes the reference ensemble comprising 10 parameter configurations. Remarkable statistical regularities were found when analyzing the perturbation landscapes. 1) In general, the D-values associated to many parameters can be highly variable; these were found to be heavily dependent on the parameter configuration tested. This observation indicates that the reproducibility of particular transcriptional readouts in the face of global perturbations is strongly dependent on the region in biochemical reaction space occupied by the signal transduction network. For example, under some parameter regimes the dynamical trajectory of the transcriptional output may be more sensitive to variation in some parameters than in others. 2) In particular, the perturbation landscape for *Tnfα *tend to exhibit and extremely rough topography, with an excess of large D-values (> 0.3) distributed heterogenously all over the surface; whereas the landscape associated to *Cxcl10 *was found to be particularly flat, with just few regions displaying large D-values (> 0.3). These findings provide convincing statistical evidence supporting the idea that the transcriptional readout of *Tnfα *should be remarkably more sensitive in the face of global fluctuations in internal reaction parameters than that expected for the transcriptional readout of *Cxcl10*. Moreover, in the case of the transcriptional readout of *Tnfα*, it is notable the way in which D-values associated to a given parameter tend to fluctuate depending on the parameter configuration, that is, the position in parameter space. Alternatively, most D-values calculated with respect to the transcriptional readout of *Cxcl10 *were found to fluctuate only slightly; in general D statistics exhibit a rather invariable tendency across different regions of parameter space. Only in few cases (specific points in biochemical reaction space) considerably large D-values were found in the landscape calculated for *Cxcl10*. For instance, the analysis reveal that only a small fraction of the whole set of reaction parameters appears to most effectively control the transcriptional readout of *Cxcl10*, which include the following parameters: *k*21*cat *(the *Dissociation Rate of IκB-NFκB*), *k*22*f *(the *Import Rate to Nucleus of NFκB*), *α*2 (Transcriptional Regulatory Strength of IRFpp* over Cxcl10), *β*2 (*Transcriptional Regulatory Strength of NFκB over Cxcl10*), *V A*2 (Cooperativity Effects of IRFpp* on Cxcl10), *V B*2 (*Cooperativity Effects of NFκB on Cxcl10*), *K β*2 (*MichaelisMenten-Constant Related to Cxcl10 *Transcription), *k*_*d*_*Cxcl *(*Degradation Rate of Cxcl10 mRNA*), *TmaxCxcl *(*Max. Transcriptional Rate of Cxcl10*), and *ρCxcl *(*Transcriptional Efficiency of the Cxcl10 Promoter*). Taken together, these simulation results point to the idea that the sensitivity/robustness of a given gene expression pattern should be strongly dependent on the architecture of the signaling fluxes influencing directly or indirectly its transcriptional activation. Following this line of arguments, it is interesting to note that the transcriptional activation of *Tnfα*, within our short time scale simulated, relies indirectly on the intranuclear activation of ERK, P38, and JNK, which activates AP1, which in turn activates the transcription of *Tnfα *along with NF*κ*B; whereas, the transcriptional activation of *Cxcl10 *only relies on NF*κ*B and IRF. Under these considerations, it should be clear that the density of signaling fluxes exerting control over the activation of *Tnfα *far exceeds the density of fluxes influencing the activation of *Cxcl10*. Our observations thus point to the idea that the propagation of multiple perturbations along the reaction cascades should differentially impact the temporal trajectory of the transcriptional readouts of *Tnfα *and *Cxcl10*. Nevertheless, such apparent differences observed in the topography of the perturbation landscapes are likely to vanish under different molecular scenarios. For example, feedback control or systematically correlated perturbations among subsets of parameters may lead to rather similar perturbation landscapes.

## Discussion

The main purpose of this *in silico *work was to explore whether important system-level attributes of a complex biomolecular network were strongly conditioned by the type of signaling tasks (i.e. particular dynamical regimes of molecular activity) simulated. Specifically, our computational approach permitted us an unbiased statistical assessment of the robustness properties, as well as the information processing capabilities, of the canonical reaction mechanism underlying TLR4-mediated signal transduction events. This was achieved by considering a broad spectrum of plausible dynamical behaviors displayed by the network (including wild type phenotypes), which are likely encountered in any cell lineage (i.e. macrophage) under diverse physiological conditions. This is the rationale behind our work, and we highlight that these considerations have been largely underappreciated in previous studies of network robustness. Recent investigations, however, have stressed the importance of assessing the spectrum of variational constraints (i.e. robustness, evolvability, epistasis, etc.) of complex developmental regulatory networks under different hypothetical and observable dynamical regimes [13,56]. Our work thus differs considerably from recent computational studies wherein heavy emphasis have been placed on the characterization of robustness of particular intracellular networks under rather limited biological circumstances [17,18,27,28].

To summarize, our numerical findings strongly suggest that the canonical TLR4 signaling network that drives crucial innate immune cellular responses in macrophages, should be operative in widely scattered regions of the biochemical reaction space; a robust property that allows the network to perform complex signaling tasks in a highly reproducible manner under rather different regimes of molecular activity, and when facing multiple kinetic uncertainties.

Deliberately, we have restricted our model signal transduction network to a simple biochemical reaction mechanism. Importantly, the design principle (topology) of the network was mathematically represented by means of basic reaction schemes defined in terms of mass action law and Hill saturation kinetics. Accordingly, information processing in our model network takes place only through the kinetic coupling of multiple, but rather simple, reaction rules accounting for ligand-receptor interaction, association and dissociation events between single or multiple reaction species, import/export fluxes between cellular compartments, enzyme-catalyzed reactions, and transcriptional control. Elaborated regulatory schemes, such as inhibitory reactions or feedback control, were not accounted for in our modeling framework. This is because within our narrow temporal window, in which immediate immune cellular responses are elicited, signal propagation is thought to be controlled in its entirety by the intrinsic crosstalking of MyD88-dependent and TRIF-dependent reaction cascades (see [46,47] and references therein). Therefore, within our simulated time window, emphasis was not placed on the complex negative feedback control arising within the NF*κ*B regulatory module, which is triggered by a wide spectrum of pro-inflammatory stimuli [57]. The many possible roles of negative feedback control deployed by the NF*κ*B regulatory module under different cellular contexts have been a central theme of investigation in intracellular signaling ([51,57]); this issue, however, was beyond the scope of our study. Nevertheless, we acknowledge that our results on the robustness properties and information processing capabilities of the TLR4 signaling network are expected to differ considerably under a different mathematical representation of the reaction topology, wherein positive/negative feedback regulation taking place at any point along the signaling cascade were accounted for. This should come as no surprise, since the crucial role of such elaborated regulatory schemes in any signal transduction system has been well documented (see for example [16,58], and references therein).

**Figure 10:**
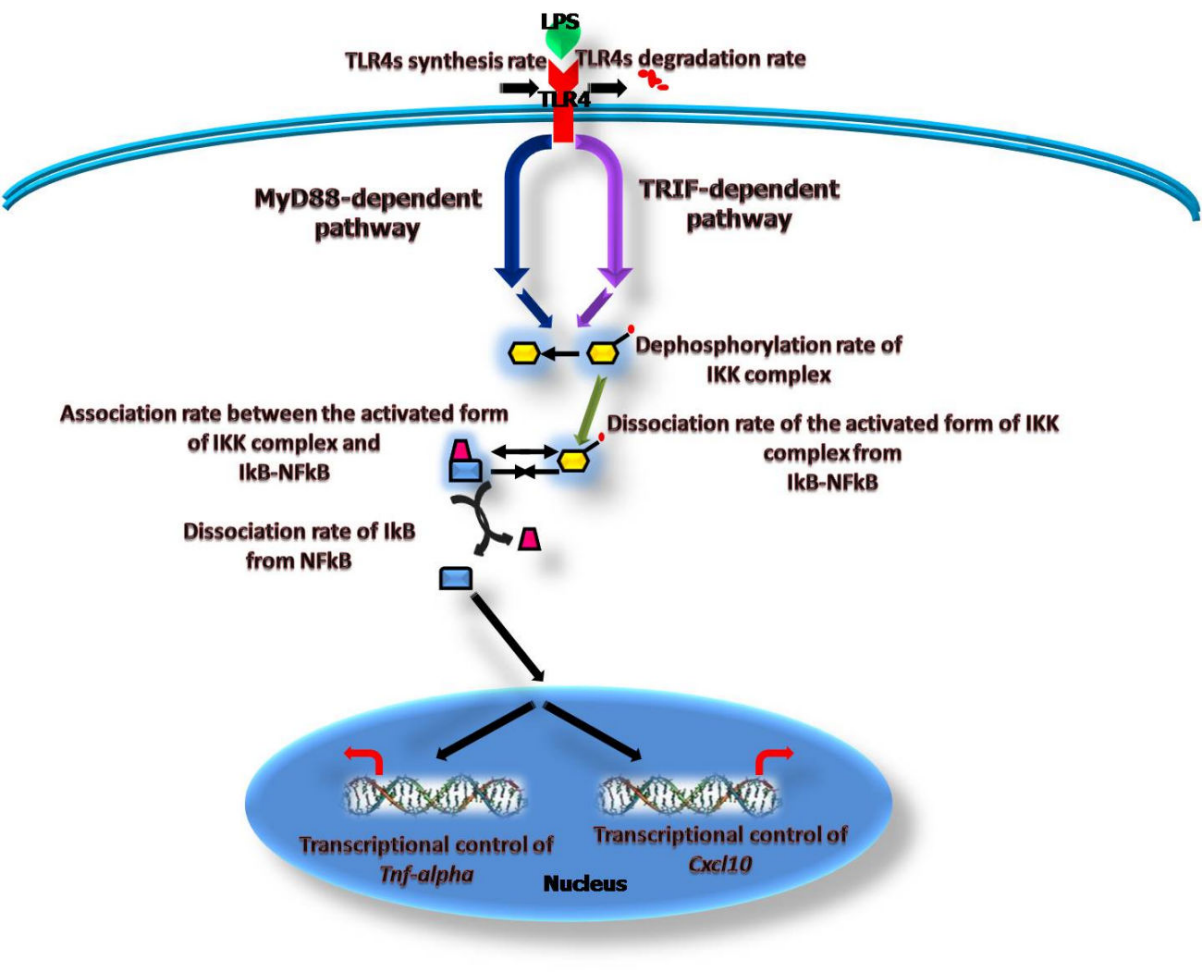
**Rate limiting steps controlling most effectively the global behavior of the canonical TLR4 signaling network**. Schematic representation on rate limiting steps inferred from non-orthogonal perturbation experiments. The analysis indicates that the global behavior of the TLR4 signaling network is most effectively control by only few reaction steps along the signaling cascades. It is inferred that global information processing in the system heavily relies on 8 biochemical reaction processes, which are quantitatively modulated by just few internal reaction parameters. For instance, the global behavior of the network was found to be remarkably sensitive to random fluctuations in the reaction parameters controlling the *Production and Degradation Rates of the TLR4 Susceptible Form *(*kps *and *kds*), the Dephosphorylation Rate of IKKcp* (*k*20), the association rate between the IKKcp* and IκB-NFκB (*k*21*f*), the dissociation rate between the IKKcp* and IκB-NFκB, (*k*21*r*), and the *Dissociation Rate of IκB-NFκB *(*k*21*cat*). In addition, the system also showed considerable sensitivity to random changes in kinetic parameteres involved in transcriptional control.

**Figure 11:**
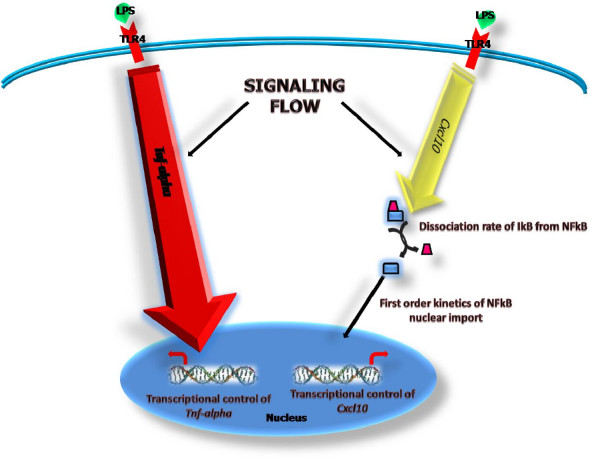
**Rate limiting steps controlling most effectively the transcriptional readouts of *Tnfα *and Cxcl10**. Schematic representations on rate limiting steps inferred from non-orthogonal perturbation experiments. The analysis indicates that the two transcriptional outputs modulated by the canonical signaling network are differentially controlled. It is inferred that the transcriptional readout of *Tnfα *is collectivelly controlled by the whole integrated reaction system; whereas the the transcriptional readout of *Cxcl10 *is most effectively controlled by only a tiny fraction of the biochemical reactions involved in signal propagation, which are quantitatively modulated by just few internal reaction parameters. For example, it was found that the *Dissociation Rate of IκB-NFκB *(*k*21*cat*), the *Import Rate to Nucleus of NFκB *(*k*22*f*), and the set of parameteres involved in transcriptional activation, seem to most effectively control this signaling ouput. The bright red arrow is to illustrate that the signaling flow that eventually leads to the transcriptional activation of *Tnfα *is tightly controlled by the whole integrated reaction network. The dull yellow arrow indicates that all reaction steps in the network, except those for which the parameters are illustrated, are not critically involved in controlling the transcriptional readout of *Cxcl10*.

Once having clarified the scope of our study, and more specifically the range of validity of our numerical experiments, we would like to discuss the biological implications of our major findings. Specifically, our global perturbation analyses provide valuable information with respect to plausible variational constraints arising in the system as a result of its canonical design principle. For example, our simulation results indicate the presence of key rate limiting steps that seem to most effectively control the dynamical behavior of the signal transduction network. In particular, statistical analyses from non-orthogonal pertubation experiments clearly show that the global behavior of the system is tightly controlled by only a tiny fraction of the reaction steps embedded in the whole reaction mechanism (see Figure 10). On the other hand, our analyses from global perturbation experiments based on the temporal profiles of transcriptional activation of the two pro-inflammatory genes modeled (*Tnfα *and *Cxcl10*) indicate two very distinct scenarios of signaling control. Firstly, the control of the transcriptional readout of *Tnfα *is surprinsingly distributed throughout the whole reaction network (see Figure 9, top panel, and Figure 11). Hence, the transcriptional activation of *Tnfα *should be tightly kinetically involved by virtue of the signaling fluxes displayed by the network upon LPS stimulation. Secondly, the control of the transcriptional readout of *Cxcl10 *was found to be sparsely distributed in the reaction network, with just few reaction steps critically involved in this signaling ouput (see Figure 9, bottom panel, and Figure 11). Taken together, our results provide mechanistic insights on complex aspects of intracellular signaling in the context of innate immune cellular responses, which might be universal principles of cellular information processing. Overall, our numerical experiments agree well with results from a recently published simulation work [20] indicating that care should be taken when analyzing the robustness properties of any biomolecular network, as these can be heavily dependent on both the quantitative outputs being evaluated, and the current kinetic status of the system (i.e. the position in biochemical reaction space). Finally, and perhaps most importantly, our computational study strongly suggests that the development of effective therapeutic strategies aimed at modulating particular cellular responses, such as metabolic fluxes, signaling and transcriptional outputs, should place heavy emphasis on the architecture of the underlying biomolecular systems. Interestingly, congruent with our findings, accumulating numerical evidence have demostrated that cellular information processing seems to emerge mainly from highly non-linear dynamics, as well as synergistic/antagonistic interactions among system's components, which can not be resolved by intuitive reasoning alone.

### Final remarks

Most systems biology studies centered on the structural and functional organization of highly-dimensional biomolecular systems point to the general idea that signal transduction networks should display the inherent capacity of accomplishing specific biological tasks in a robust manner (see [12] and references therein). Robustness seems to be a natural property stemming from the evolved design principle of biomolecular networks [7-9], which allow them to inhabit sloppy parameter spaces wherein system's behavior turn out to be highly sensitive to variation along a few stiff directions, while being remarkably insensitive to variation along a large number of sloppy axes in parameter space [11,30]. Notably, accurate computational reconstructions of experimentally reported dynamical behaviors of many signal transduction networks have been successfully achieved [20,51,55,57]. Interestingly, standard mathematical representations of the reaction topology of most signaling network models are typically founded on highly non-linear, but relatively simple, biochemical reaction rules, which despite being an abvious simplification of the underlying biochemistry have proven successful at providing mechanistic insight [20,51,55,57]. This is an intriguing observation from an evolutionary standpoint. This suggests, for example, that the underlying mathematical structure of most signal transduction networks that has been favored over evolution to process in an efficient and robust manner the biochemical information arising in the cell, might simply rely on basic dynamic rules ([59,60]). Intuitively, the most variable component affecting the temporal variation in the activity of the molecular species involved in a certain signaling event would be the number of the contributing reaction velocities to a particular flux. Following this line of arguments, it is tempting to speculate on the possibility that the deterministic component of the dynamical trajectories displayed by most signaling networks might have been the result of selection for simple biochemical reaction rules built, for example, upon mass action and Hill-like saturation kinetics.

## Models and computational framework

The mathematical representation of the canonical reaction network retrieved from the literature, and the whole set of numerical experiments that are described below were implemented in Mathematica^® ^6.0.

### Mathematical formulation of the signal transduction network in the language of dynamical systems

A signal transduction network can be appropriately conceived in dynamical terms, whose internal regulatory schemes, reaction rules and associated control parameters underlying the trajectories of the system can be formulated, as a first approximation, via basic principles of biochemical reaction. Ordinary Differential Equations-based models grounded on mass action law (first and second reaction kinetic orders) and Hill saturation kinetics, provide a suitable macroscopic approximation to intracellular signal transduction dynamics (fluxes), as well as transcriptional phenomena. Under this modeling formalism, a biochemical reaction network can be described on the basis of the state space formulation with the following mathematical constructs:

1 A network with *n *reaction species is represented by the state vector:(1)

In our case, the TLR4 signaling network model incorporates *n *= 76 reaction species, including receptors, adapters, kinases, transcription factors and mRNAs.

2 The biochemical reaction parameters controlling the signaling flux through the network and transcriptional processes of target genes are incorporated in the reaction vector(2)

With *m *= 116 internal reaction coefficients for the TLR4 network. Here, Θ encompasses a wide spectrum of parameters of different biochemical nature, ranging from transition rates between receptor states (susceptible ⇌ activated), production and degradation rates of receptors, association/dissociation rates among intracelular molecular species, phospho/dephosphorylation rates, nuclear import/export rates, maximal transcriptional rates, transcriptional efficiencies, Michaeles-Menten constants, cooperative coefficients, and mRNA degradation rates (see Additional file 1 for a detailed description of these parameters and their assigned range of values).

According to the above mathematical expressions, the state space non-linear representation of the biochemical reaction network is thus given by:(3)

Where *f*(·) defines a non-linear state transition function accounting for reaction velocities or fluxes (see Additional file 1 for a detailed description of the dynamical system), which can be grounded on mass action laws and/or Hill kinetics, according to the reaction mechanisms modeled (receptor activation kinetics, binding and enzymatic reactions, or transcriptional dynamics). **Y**_0 _represents the vector of initial concentrations for the reaction species at time *t*_0_; *g*(·) defines a measurement function which is solved numerically; whereas **X **∈ ℜ^*n *^gives the measurement output vector representing the concentration of the reaction species at a given point in time. In our case, the TLR4 signaling network model accounts for 76 dynamic variables (reaction species, *Y*_*j *_∀ *j *∈ (1, 2, 3, ....,76)), 32 of which were assigned zero initial conditions (see Additional file 1).

### Multiparametric sensitivity analysis (MPSA): a combination of uncertainty and sensitivity analyses

The MPSA approach was first introduced by Hornberger and Chang [61,62]. This is a computational strategy specially suitable for characterizing the relative importance of the parameters of a multidimensional mathematical model. Additionally, a MPSA also provides the means for the identification of possible correlations within the system under study. The basic idea of the MPSA method is to generate ensembles of different input parameter configurations for systematically evaluating the range of responses (outputs) accessible to a given mathematical model. This can be achieved via Monte Carlo simulations, in which the model is run iteratively using sets of parameters drawn randomly from pre-specified distributions. Since the natural distributions of parameter values for actual biological networks are completely unknown, we thus implemented uniform probability distributions in our simulations. Since our biochemical reaction network inhabits a multidimensional parameter space encompassing 116 biochemical reaction axes, which needs to be sampled efficiently, we thus implemented a Latin Hypercube Sampling (LHS) method so as to generate representative ensembles of different parameter configurations in both the immediate and distant vicinity of a reference point in parameter space. This initial approach is aimed at injecting uncertainty into the model's inputs, and has thus been coined the term *Uncertainty Analysis *[23]. We then made use of these ensembles of parameter configurations obtained from previously assembled reference points in parameter space, to statistically characterize the robust properties of the canonical reaction topology underlying TLR4-mediated signaling processes in macrophages; a computational approach known as *Sensitivity Analysis *[23-26]. We define a decision rule that allowed for the classification of each model evaluation as either acceptable (robust) or unacceptable (nonrobust). Further, statistical analyses, based on Kolmogorov-Smirnov (KS) tests, were performed on the occurences of acceptable and unacceptable cases, which are summarized for each model parameter. The larger the difference between the cumulative frequencies of the two cases, which is reflected by large values of the D statistic obtained from KS tests, the more significant is a given parameter. The MPSA method can be summarized in the following steps:

1 Selection of reference parameter configurations (vectors) to be perturbed.

2 Set a relatively large range of variation for each model parameter in order to account for a wide spectrum of biologically plausible perturbations (i.e. single or combined mutations, thermal fluctuations, etc.). In our case, a perturbation variable, *ρ*, was sampled in this way: *ρ *~ *U*(-1, 1); a perturbation function was then applied over a reference parameter *i*, *θ*_*i*, *ref*_, in order to obtain a newly perturbed parameter *θ*_*i*, *pert *_= 10^ρ ^* *θ*_*i*, *ref*_

3 Under this perturbation strategy we initially generated seeds of LHS matrices with the basic structure as illustrated in Table 3, wherein a row vector stands for a perturbed parameter configuration. In this way, the matrix shown represents a set of input vectors distributed in parameter space in the vicinity (either immediate or distant) of a previously defined reference point; this matrix is assembled via the LHS strategy, and is designed for the systematic evaluation of the model's output. Note that in our case 5000 parameter configurations were generated from a previously defined reference point in parameter space. We constructed ensembles of 100 LHS matrices for evaluating the robust information processing capabilities of the network in different regions of biochemical reaction space. We also constructed an ensemble of 10 LHS matrices in order to test for the robustness properties of the two experimentally reported transcriptional outputs of the network. Before simulating each previously assembled seed LHS matrix, however, we permuted the elements of each column of a matrix as illustrated in Table 4. In this way, permuting the matrices permitted us to avoid any kind of bias in model evaluations.

**Table 3:** A seed LHS matrix. IPPC stands for initial perturbed parameter configuration. *θ*_*i *_indicates any reference parameter value *i *to be perturbed systematically

IPPC	*θ*_*i*_	*θ*_1, *ref*_	*θ*_2, *ref*_	ℭ	⋯	*θ*_116, *ref*_
*Vector*	Θ^1^			⋯	⋯	

*Vector*	Θ^2^			⋯	⋯	

*Vector*	Θ^3^			⋯	⋯	

*Vector*	Θ^4^			⋯	⋯	

*Vector*	Θ^5^			⋯	⋯	

*Vector*	Θ^6^			⋯	⋯	

*Vector*	Θ^7^			⋯	⋯	

⋮		⋮	⋮	⋮	⋮	⋮

⋮		⋮	⋮	⋮	⋮	⋮

*Vector*	Θ^5000^			⋯	⋯	

**Table 4:** A permuted version of the seed LHS matrix. NAPC stands for newly assembled parameter configuration. *θ*_*i *_indicates any reference parameter value *i *to be perturbed systematically

NAPC	*θ*_*i*_	*θ*_1_	*θ*_2_	⋯	⋯	*θ*_116_
*Vector*	Θ^1^*			⋯	⋯	

*Vector*	Θ^2^*			⋯	⋯	

*Vector*	Θ^3^*			⋯	⋯	

*Vector*	Θ^4^*			⋯	⋯	

*Vector*	Θ^5^*			⋯	⋯	

*Vector*	Θ^6^*			⋯	⋯	

*Vector*	Θ^7^*			⋯	⋯	

⋮		⋮	⋮	⋮	⋮	⋮

⋮		⋮	⋮	⋮	⋮	⋮

*Vector*	Θ^5000^*			⋯	⋯	

4 Each LHS matrix was then simulated, and the corresponding discrepancy function evaluated, which is of the form:(5)

With *J *denoting a reference dynamical trajectory with associated parameter configuration Θ_*ref*_, and Θ_*pert *_being a perturbed version of it obtained from a LHS matrix; with *h *∈ (1, ..., 5000) being any evaluation of the discrepancy function.

5 This step implied determining whether a given perturbed parameter configuration was acceptable (robust) or unacceptable (sensitive) by comparing the discrepancy function value to a given threshold. If the discrepancy function value was found to be below the threshold value the evaluated parameter configuration was then classified as acceptable; otherwhise it was classified as unacceptable. Previous computational works suggest that results from MPSA should not be affected considerably with the choice of a given discrepancy function [25,53,63]. Here we implemented the average of the discrepancy function as our threshold value, defined as follows:(6)

6 After systematic evaluation of the discrepancy function for each LHS matrix, statistical assessment followed in order to determine whether a given parameter configuration was deemed either acceptable or unacceptable. To do this, we applied the KS test to assess the global sensitivity of the system's output with respect to perturbations targeting individual parameters. The KS test provides the means for evaluating the cumulative frequency of the observations (parameter values) as a function of class, and calculate the maximum vertical distance between cumulative frequency distribution curves for *m *acceptable and *n *unacceptable cases of any given parameter *θ*_*j*_. This is obtained by calculating the D statistic, which is defined in this way:(7)

Where *S*(*θ*_*j*_) and  represent the cumulative frequency functions corresponding to acceptable and unacceptable cases, respectively, with *θ*_*j *_being any reaction parameter of the signal transduction network. Importantly, this estimator provides a robust quantitative notion for the sensitivity/robustness of the network model to random perturbations of the reaction parameters. The higher the D-value the more sensitive is the dynamical behavior of the network model with respect to variation of a given parameter, when the remaining parameters (i.e. biochemical background of the network) are also varied.

### Total parameter variation

The total parameter variation estimator provides a quantitative notion of the order of magnitude in the variation of a perturbed parameter configuration obtained from a reference one. This estimator is defined as:(8)

We calculated all *T *values for those perturbed parameter configurations that were deemed either robust or fragile obtained from a reference parameter configuration, Θ_*ref*_, exhibiting a global dynamical trajectory *J *(∀ *J *∈ (1, ......., 100))

### Local and global perturbation analysis of input-output maps

Here, it is described the global (non-orthogonal) and local (orthogonal) perturbation strategies with respect to particular inputs of the network. Specifically, we assessed the reproducibility of the network's transcriptional outputs (*Tnfα *and *Cxcl10*) in the face of single (local) and multiple simulataneous (global) perturbations targeting the internal reaction parameters of the network. Local perturbations were quantified via the following metric:(9)

This can be referred to as an "Overal Senstitivity Index" [17,55], being *s*_*i*_(*t*) defined as follows:(10)

With the variable *Output *representing either *Tnfα *or *Cxcl10*, and *θ*_*j *_giving any internal reaction parameter *j *belonging to a reference parameter configuration Θ_*ref*_. In this local perturbation experiment we focused only on those reaction parameters accounting for signaling fluxes from the cell membrane surface down to the nucleus; that is to say, perturbations targeting transcriptional parameters were not simulated. On the other hand, to perform global perturbation analysis based on the effects of multiple induced perturbations at the level of transcriptional readouts, we implemented the same MPSA described above. However, in this case we followed a different perturbation scheme. We chose a perturbation variable, *ρ*, to be sampled in this way: *ρ *~ *U*(- 1, 1); a perturbation function was then applied over a reference parameter *i*, *θ*_*i*, *ref*_, in order to obtain a newly perturbed parameter *θ*_*i*, *pert *_= 5^ρ ^* *θ*_*i*, *ref *_. In this experiment, we perturbed the entire biochemical reaction space, including internal reaction parameters related to both signaling fluxes and transcriptional processes, and assessed the effects of the perturbations at the level of each transcriptional readout separately (*Tnfα *or *Cxcl10*), via the following discrepancy function:(11)

This time, our criterion used to categorize a parameter configuration as acceptable or unacceptable was based on the following threshold value:(12)

Given in arbitrary units of discrepancy, this threshold was selected upon a detailed analysis of both the qualitative and quantitative effects of the perturbations on the temporal dynamics of the transcriptional readouts.

## Competing interests

The authors declare that they have no competing interests.

## Authors' contributions

JG designed the study, developed the computational framework, performed simulations and statistical analysis, discussed the results and drafted the manuscript. GSL and SUI discussed the results and helped to draft the manuscript. All authors read and approved the final version of the manuscript.

## Supplementary Material

Additional file 1 **Mathematical structure of the signal transduction network: kinetic parameters, initial conditions, and rate equations**. This file contains a detailed description of our modeling framework. Ranges of values for kinetic parameters and initial conditions are given, which were selected according to several computational strategies described in the main text. Our system of rate equations implemented for simulating intracellular fluxes and propagation of kinetic uncertainties through the TLR4 signal transduction network, is also described. Click here for file
