# Supplementary material for: Propagation of kinetic uncertainties through a canonical topology of the TLR4 signaling network in different regions of biochemical reaction space
Source: Theor Biol Med Model. 2010 Mar 15;7:7. doi: 10.1186/1742-4682-7-7 (PMC2907738; doi:10.1186/1742-4682-7-7)
Supplement: Additional file 1 — Mathematical structure of the signal transduction network: kinetic parameters, initial conditions, and rate equations. This file contains a detailed description of our modeling framework. Ranges of values for kinetic parameters and initial conditions are given, which were selected according to several computational strategies described in the main text. Our system of rate equations implemented for simulating intracellular fluxes and propagation of kinetic uncertainties through the TLR4 signal transduction network, is also described. [file 1742-4682-7-7-S1.PDF]

Propagation of kinetic uncertainties through a canonical topology of the TLR4 signaling network in different regions of biochemical reaction space

Jayson Gutiérrez, Georges St Laurent III, Silvio Urcuqui-Inchima

Additional file 1 — Mathematical structure of the signal transduction network: kinetic parameters, initial conditions, and rate equations

Mathematical structure of the signal transduction network

The reaction rules governing the dynamical trajectory of each reaction species in the TLR-4-mediated signaling network were modeled via mass action law principles of first and second order for the processes involved solely in intracellular signaling fluxes. Here, reaction rules were assumed to be governed by either a binding (resulting in molecular activation or deactivation) or an enzymatic reaction. For example, for a unimolecular reaction involving any molecular species A the reaction velocity was then formulated as $r = k * [A]$, where $[A]$ stands for the average concentration, over an ensemble of cells (i.e. a cell culture), of A, and k indicating a kinetic coefficient. Key molecular processes were thus modeled according to this simple reaction rule, including non-specific degradation processes of single species, dissociation of molecular complexes, as well as diffusion of species between cellular compartments (see below). In the case of biomolecular reactions involving any pair of molecular species A and B, we implemented the following simple rule to approximate the reaction velocity: $r = k * [A] * [B]$; this reaction rule was implemented in the case of binding/association reactions. On the other hand, ligand-receptor kinetics and transcriptional processes were modeled via Hill saturation kinetics and some generalizations of these kinetics (see below).

Kinetic coefficients involved in unimolecular and bimolecular reactions were sampled from uniform distributions ranging on [0,2.5]. Reaction parameters related with Hill saturation kinetics, such as Michaelis-Menten constants, were sampled from the uniform distribution ranging on [0.5,225], whereas Hill (cooperative) coefficients were sampled on [0.5,10]. Maximal transcriptional rates and transcriptional efficiencies were sampled from uniform distributions ranging on [0.5,25] and [0,1], respectively (see below). Uniform distributions were assumed because information on the possible distribution of reaction parameters for this signal transduction has not been previously reported.

Most reaction species in the network were assigned non-zero initial concentrations, whose values were sampled from the uniform distribution ranging on [0,1], with some reaction species being assigned zero initial values based on biological intuition. For example, in the case of molecular complexes or phosphorylated forms and mRNA species the concentrations were set to 0, which amounts to 32 zero initial conditions in the network model (see below).

Kinetic parameters

Supplementary Table 1 - Reaction parameters, and their corresponding biochemical properties, used for simulating the ensemble of 100 dynamical trajectories.

Reaction Parameter	Biochemical Property	Range
kps	Production Rate of the TLR4 Susceptible Form	$[6.0\text{x}10^{-4}, 0.0995]$ (nM h^{-1})
kds	Degradation Rate of the TLR4 Susceptible Form	$[8.8\text{x}10^{-6}, 0.0990]$ (h^{-1})
ksa	Transition Rate between Susceptible-Activated TLR4	$[5.8\text{x}10^{-4}, 0.0976]$ (s^{-1})
kas	Transition Rate between Activated-Susceptible TLR4	$[2.0\text{x}10^{-4}, 0.0980]$ (s^{-1})
kda	Degradation Rate of TLR4 Activated Form	$[5.0\text{x}10^{-5}, 0.0996]$ (h^{-1})
K_b	MichaelisMenten-Constant Related to TLR4-Activation	$[0.0218, 39.9855]$ (nM)
n	Cooperativity Coefficient Related to TLR4-Activation	$[0.0585, 9.9587]$
$k1f$	Association between MyD88Mal + TLR4	$[5.0\text{x}10^{-5}, 0.0995]$ ($nM^{-1}s^{-1}$)
$k1r$	Dissociation of MyD88Mal-TLR4	$[1.0\text{x}10^{-3}, 0.0988]$ (s^{-1})
$k2f$	Association between MyD88Mal-TLR4 + IRAK4	$[1.0\text{x}10^{-3}, 0.0980]$ ($nM^{-1}s^{-1}$)
$k2r$	Dissociation of MyD88Mal-TLR4-IRAK4	$[1.1\text{x}10^{-3}, 0.0994]$ (s^{-1})
$k3$	Dephosphorylation Rate of IRAK4p*	$[3.8\text{x}10^{-3}, 0.0998]$ (s^{-1})
$k4f$	Association between IRAK4p* + IRAK1	$[5.9\text{x}10^{-5}, 0.0992]$ ($nM^{-1}s^{-1}$)
$k4r$	Dissociation of IRAK4p*-IRAK1	$[2.3\text{x}10^{-3}, 0.0982]$ (s^{-1})
$k5$	Dephosphorylation Rate of IRAK1p*	$[1.2\text{x}10^{-3}, 0.0988]$ (s^{-1})
$k6f$	Association between IRAK1p* + TRAF6	$[2.0\text{x}10^{-3}, 0.0963]$ ($nM^{-1}s^{-1}$)
$k6r$	Dissociation of IRAK1p*-TRAF6	$[4.9\text{x}10^{-4}, 0.0989]$ (s^{-1})
$k7f$	Association between IRAK1p*-TRAF6 + TABTAK	$[3.3\text{x}10^{-4}, 0.0981]$ ($nM^{-1}s^{-1}$)
$k7r$	Dissociation of IRAK1p*-TRAF6-TABTAK	$[1.8\text{x}10^{-3}, 0.0987]$ (s^{-1})
$k8$	Dephosphorylation Rate of TABTAKp*	$[3.7\text{x}10^{-4}, 0.0985]$ (s^{-1})
$k9f$	Association between TABTAKp* + MKK4/7	$[2.3\text{x}10^{-4}, 0.0999]$ ($nM^{-1}s^{-1}$)
$k9r$	Dissociation of TABTAKp*-MKK4/7	$[1.6\text{x}10^{-5}, 0.0992]$ (s^{-1})
$k10$	Dephosphorylation Rate of MKK4/7p*	$[2.1\text{x}10^{-3}, 0.0965]$ (s^{-1})
$k11f$	Association between MKK4/7p* + JNK	$[1.3\text{x}10^{-3}, 0.0996]$ ($nM^{-1}s^{-1}$)
$k11r$	Dissociation of MKK4/7p*-JNK	$[1.6\text{x}10^{-3}, 0.0975]$ (s^{-1})
$k12$	Dephosphorylation Rate of JNKp*	$[7.4\text{x}10^{-4}, 0.0977]$ (s^{-1})
$k13f$	Import Rate to Nucleus of JNKp*	$[3.4\text{x}10^{-3}, 0.0998]$ ($nM^{-1}s^{-1}$)
$k13r$	Export Rate from Nucleus of JNKp*n	$[1.6\text{x}10^{-4}, 0.0988]$ (s^{-1})
$k14f$	Association between TABTAKp* + MKK3/6	$[2.5\text{x}10^{-3}, 0.0961]$ ($nM^{-1}s^{-1}$)
$k14r$	Dissociation of TABTAKp*-MKK3/6	$[2.9\text{x}10^{-3}, 0.0989]$ (s^{-1})
$k15$	Dephosphorylation Rate of MKK3/6p*	$[1.3\text{x}10^{-3}, 0.0993]$ (s^{-1})
$k16f$	Association between MKK3/6p* + p38	$[2.2\text{x}10^{-3}, 0.0993]$ ($nM^{-1}s^{-1}$)
$k16r$	Dissociation of MKK3/6p*-p38	$[7.9\text{x}10^{-6}, 0.0997]$ (s^{-1})
$k17$	Dephosphorylation Rate of p38p*	$[2.9\text{x}10^{-4}, 0.0985]$ (s^{-1})
$k18f$	Import Rate to Nucleus of p38p*	$[2.5\text{x}10^{-3}, 0.0971]$ ($nM^{-1}s^{-1}$)
$k18r$	Export Rate from Nucleus of p38p*n	$[7.7\text{x}10^{-4}, 0.0990]$ (s^{-1})
$k19f$	Association between TABTAKp* + IKKc	$[1.2\text{x}10^{-4}, 0.0996]$ ($nM^{-1}s^{-1}$)
$k19r$	Dissociation of TABTAKp*-IKKc	$[1.0\text{x}10^{-4}, 0.0993]$ (s^{-1})
$k20$	Dephosphorylation Rate of IKKcp*	$[1.4\text{x}10^{-3}, 0.0996]$ (s^{-1})
$k21f$	Association between IKKcp* + IκB-NFκB	$[7.3\text{x}10^{-4}, 0.0999]$ ($nM^{-1}s^{-1}$)
$k21r$	Dissociation of IKKcp*-IκB-NFκB	$[1.1\text{x}10^{-3}, 0.0997]$ (s^{-1})
$k22f$	Import Rate to Nucleus of NFκB	$[1.5\text{x}10^{-3}, 0.0985]$ ($nM^{-1}s^{-1}$)
$k22r$	Export Rate from Nucleus of NFκBn	$[1.3\text{x}10^{-3}, 0.0994]$ (s^{-1})
$k23f$	Association between IKKcp* + TpL2	$[4.9\text{x}10^{-4}, 0.0997]$ ($nM^{-1}s^{-1}$)
$k23r$	Dissociation of IKKcp*-TpL2	$[1.5\text{x}10^{-4}, 0.0992]$ (s^{-1})
$k24$	Dephosphorylation Rate of TpL2p*	$[1.9\text{x}10^{-3}, 0.0987]$ (s^{-1})
$k25f$	Association between TpL2p* + MKK1/2	$[1.2\text{x}10^{-5}, 0.0998]$ ($nM^{-1}s^{-1}$)
$k25r$	Dissociation of TpL2p*-MKK1/2	$[2.6\text{x}10^{-4}, 0.0995]$ (s^{-1})
$k26$	Dephosphorylation Rate of MKK1/2p*	$[1.1\text{x}10^{-4}, 0.0991]$ (s^{-1})
$k27f$	Association between MKK1/2p* + ERK	$[2.1\text{x}10^{-3}, 0.0987]$ ($nM^{-1}s^{-1}$)
$k27r$	Dissociation of MKK1/2p*-ERK	$[1.0\text{x}10^{-3}, 0.0987]$ (s^{-1})
$k28$	Dephosphorylation Rate of ERKp*	$[2.0\text{x}10^{-3}, 0.0987]$ (s^{-1})
$k29f$	Import Rate to Nucleus of ERKp*	$[8.5\text{x}10^{-4}, 0.0981]$ ($nM^{-1}s^{-1}$)
$k29r$	Export Rate from Nucleus of ERKp*n	$[3.2\text{x}10^{-3}, 0.0995]$ (s^{-1})
$k30f$	Association between TLR4 + I_1	$[1.4\text{x}10^{-3}, 0.0987]$ ($nM^{-1}s^{-1}$)
$k30r$	Dissociation of TLR4-I_1	$[1.6\text{x}10^{-3}, 0.0965]$ (s^{-1})
$k31f$	Association between TLR4-I_1 + I_2	$[2.1\text{x}10^{-4}, 0.0998]$ ($nM^{-1}s^{-1}$)
$k31r$	Dissociation of TLR4-I_1-I_2	$[9.5\text{x}10^{-4}, 0.0997]$ (s^{-1})

Supplementary Table 2 - Reaction parameters, and their corresponding biochemical properties, used for simulating the ensemble of 100 dynamical trajectories.

Reaction Parameter	Biochemical Property	Range
$k32f$	Association between TLR4-I_1-I_2 + I_3	$[9.3\text{x}10^{-4}, 0.0986]$ ($nM^{-1}s^{-1}$)
$k32r$	Dissociation of TLR4-I_1-I_2-I_3	$[3.7\text{x}10^{-4}, 0.0977]$ (s^{-1})
$k33f$	Association between TLR4-I_1-I_2-I_3 + TRAM	$[2.4\text{x}10^{-3}, 0.0999]$ ($nM^{-1}s^{-1}$)
$k33r$	Dissociation of TLR4-I_1-I_2-I_3-TRAM	$[6.7\text{x}10^{-5}, 0.0996]$ (s^{-1})
$k34f$	Association between TLR4-I_1-I_2-I_3-TRAM + TRIF	$[4.6\text{x}10^{-4}, 0.0996]$ ($nM^{-1}s^{-1}$)
$k34r$	Dissociation of TLR4-I_1-I_2-I_3-TRAM-TRIF	$[1.2\text{x}10^{-3}, 0.0998]$ (s^{-1})
$k35f$	Association between TLR4-I_1-I_2-I_3-TRAM-TRIF + RIP1	$[1.6\text{x}10^{-3}, 0.0993]$ ($nM^{-1}s^{-1}$)
$k35r$	Dissociation of TLR4-I_1-I_2-I_3-TRAM-TRIF-RIP1	$[2.1\text{x}10^{-3}, 0.0992]$ (s^{-1})
$k36f$	Association between TLR4-I_1-I_2-I_3-TRAM-TRIF-RIP1 + AIP1	$[2.7\text{x}10^{-4}, 0.0980]$ ($nM^{-1}s^{-1}$)
$k36r$	Dissociation of TLR4-I_1-I_2-I_3-TRAM-TRIF-RIP1-AIP1	$[1.6\text{x}10^{-3}, 0.0996]$ (s^{-1})
$k37f$	Association between TLR4-I_1-I_2-I_3-TRAM-TRIF-RIP1-AIP1 + TRAF6	$[6.1\text{x}10^{-4}, 0.0985]$ ($nM^{-1}s^{-1}$)
$k37r$	Dissociation of TLR4-I_1-I_2-I_3-TRAM-TRIF-RIP1-AIP1-TRAF6	$[2.3\text{x}10^{-3}, 0.0999]$ (s^{-1})
$k38f$	Association between TLR4-I_1-I_2-I_3-TRAM-TRIF + TRAF6	$[3.2\text{x}10^{-3}, 0.0991]$ ($nM^{-1}s^{-1}$)
$k38r$	Dissociation of TLR4-I_1-I_2-I_3-TRAM-TRIF-TRAF6	$[3.1\text{x}10^{-3}, 0.0999]$ (s^{-1})
$k39f$	Association between TLR4-I_1-I_2-I_3-TRAM-TRIF + TBK1	$[5.0\text{x}10^{-4}, 0.0996]$ ($nM^{-1}s^{-1}$)
$k39r$	Dissociation of TLR4-I_1-I_2-I_3-TRAM-TRIF-TBK1	$[3.1\text{x}10^{-4}, 0.0989]$ (s^{-1})
$k40f$	Association between TLR4-I_1-I_2-I_3-TRAM-TRIF-TBK1 + IRF	$[9.1\text{x}10^{-4}, 0.0991]$ ($nM^{-1}s^{-1}$)
$k40r$	Dissociation of TLR4-I_1-I_2-I_3-TRAM-TRIF-TBK1-IRF	$[4.1\text{x}10^{-4}, 0.0997]$ (s^{-1})
$k41$	Dephosphorylation Rate of IRFp*	$[5.7\text{x}10^{-4}, 0.0996]$ (s^{-1})
$k42f$	Dimerization of IRFp* with IRFp*	$[5.3\text{x}10^{-4}, 0.0998]$ ($nM^{-1}s^{-1}$)
$k42r$	Dissociation of IRFpp*	$[9.1\text{x}10^{-4}, 0.0998]$ (s^{-1})
$k43f$	Export Rate to Nucleus of IRFpp*	$[3.5\text{x}10^{-3}, 0.0994]$ ($nM^{-1}s^{-1}$)
$k43r$	Import Rate from Nucleus of IRFpp*n	$[5.5\text{x}10^{-4}, 0.0978]$ (s^{-1})
$k44f$	Association between JNKp*n and AP1n	$[1.1\text{x}10^{-3}, 0.0990]$ ($nM^{-1}s^{-1}$)
$k44r$	Dissociation of JNKp*n-AP1n	$[4.6\text{x}10^{-4}, 0.0987]$ (s^{-1})
$k45f$	Association between p38p*n and AP1n	$[4.5\text{x}10^{-3}, 0.0994]$ ($nM^{-1}s^{-1}$)
$k45r$	Dissociation of p38p*n-AP1n	$[8.8\text{x}10^{-4}, 0.0999]$ (s^{-1})
$k46f$	Association between ERKp*n and AP1n	$[7.3\text{x}10^{-4}, 0.0982]$ ($nM^{-1}s^{-1}$)
$k46r$	Dissociation of ERKp*n-AP1n	$[2.8\text{x}10^{-4}, 0.0991]$ (s^{-1})
$k2cat$	Phosphorylation Rate of IRAK4	$[1.0\text{x}10^{-4}, 0.0998]$ (s^{-1})
$k4cat$	Phosphorylation Rate of IRAK1	$[1.8\text{x}10^{-4}, 0.0997]$ (s^{-1})
$k7cat$	Phosphorylation Rate of TABTAK	$[1.9\text{x}10^{-3}, 0.0999]$ (s^{-1})
$k9cat$	Phosphorylation Rate of MKK4/7	$[2.5\text{x}10^{-3}, 0.0993]$ (s^{-1})
$k11cat$	Phosphorylation Rate of JNK	$[5.4\text{x}10^{-5}, 0.0999]$ (s^{-1})
$k14cat$	Phosphorylation Rate of MKK3/6	$[9.8\text{x}10^{-4}, 0.0989]$ (s^{-1})
$k16cat$	Phosphorylation Rate of p38	$[3.2\text{x}10^{-3}, 0.0997]$ (s^{-1})
$k19cat$	Phosphorylation Rate of IKKc	$[8.8\text{x}10^{-4}, 0.0999]$ (s^{-1})
$k21cat$	Dissociation Rate of IκB-NFκB	$[7.5\text{x}10^{-4}, 0.0991]$ (s^{-1})
$k23cat$	Phosphorylation Rate of TpL2	$[5.3\text{x}10^{-4}, 0.0999]$ (s^{-1})
$k25cat$	Phosphorylation Rate of MKK1/2	$[3.8\text{x}10^{-4}, 0.0993]$ (s^{-1})
$k27cat$	Phosphorylation Rate of ERK	$[1.5\text{x}10^{-3}, 0.0994]$ (s^{-1})
$k40cat$	Phosphorylation Rate of IRF	$[0.5049, 0.9959]$ (s^{-1})
$\alpha 1$	Transcriptional Strength of AP1 over *Tnfα*	$[0.0745, 3.9572]$
$\beta 1$	Transcriptional Strength of NFκB over *Tnfα*	$[0.1313, 3.8909]$
$VA1$	Cooperativity Effects of AP1 on *Tnfα* Transcription	$[5.0094, 9.9702]$
$VB1$	Cooperativity Effects of NFκB on *Tnfα* Transcription	$[1.3\text{x}10^{-3}, 0.0994]$
$K\beta 1$	MichaelisMenten-Constant Related to *Tnfα* Transcription	$[0.5178, 0.9963]$ (nM)
k_dTnf	Degradation Rate of *Tnfα* mRNA	$[0.5012, 0.9973]$ (h^{-1})
$\alpha 2$	Transcriptional Strength of IRFpp* over *Cxcl10*	$[3.9\text{x}10^{-2}, 3.9763]$
$\beta 2$	Transcriptional Strength of NFκB over *Cxcl10*	$[5.9\text{x}10^{-2}, 3.9571]$
$VA2$	Cooperativity Effects of IRFpp* on *Cxcl10*	$[7.1\text{x}10^{-4}, 0.0999]$
$VB2$	Cooperativity Effects of NFκB on *Cxcl10*	$[7.6\text{x}10^{-4}, 0.0987]$
$K\beta 2$	MichaelisMenten-Constant Related to *Cxcl10* Transcription	$[5.0305, 9.9171]$ (nM)
k_dCxc	Degradation Rate of *Cxcl10* mRNA	$[5.6\text{x}10^{-4}, 0.0997]$ (h^{-1})
$TmaxTnf$	Max. Transcriptional Rate of *Tnfα*	$[1.0033, 1.9856]$ (nM h^{-1})
$TmaxCxc$	Max. Transcriptional Rate of *Cxcl10*	$[1.0073, 1.9859]$ (nM h^{-1})
ρTnf	Transcriptional Efficiency of the *Tnfα* Promoter	$[2.0\text{x}10^{-3}, 0.0996]$
ρCxc	Transcriptional Efficiency of the *Cxcl10* Promoter	$[2.3\text{x}10^{-5}, 0.0975]$

Supplementary Table 3 - Reaction parameters, and their corresponding biochemical properties, used for simulating the ensemble of 10 predictive dynamical trajectories.

Reaction Parameter	Biochemical Property	Range
kps	Production Rate of the TLR4 Susceptible Form	$[8.8\text{x}10^{-3}, 0.8037]$ (nM h^{-1})
kds	Degradation Rate of the TLR4 Susceptible Form	$[4.8\text{x}10^{-3}, 0.8258]$ (h^{-1})
ksa	Transition Rate between Susceptible-Activated TLR4	$[5.5\text{x}10^{-3}, 0.0929]$ (s^{-1})
kas	Transition Rate between Activated-Susceptible TLR4	$[1.0\text{x}10^{-2}, 0.0944]$ (s^{-1})
kda	Degradation Rate of TLR4 Activated Form	$[3.4\text{x}10^{-3}, 0.2487]$ (h^{-1})
K_b	MichaelisMenten-Constant Related to TLR4-Activation	$[2.0564, 224.3379]$ (nM)
n	Cooperativity Coefficient Related to TLR4-Activation	$[0.5070, 7.4609]$
$k1f$	Association between MyD88Mal + TLR4	$[5.5\text{x}10^{-4}, 0.0937]$ ($nM^{-1}s^{-1}$)
$k1r$	Dissociation of MyD88Mal-TLR4	$[3.3\text{x}10^{-3}, 0.0964]$ (s^{-1})
$k2f$	Association between MyD88Mal-TLR4 + IRAK4	$[2.1\text{x}10^{-2}, 0.0645]$ ($nM^{-1}s^{-1}$)
$k2r$	Dissociation of MyD88Mal-TLR4-IRAK4	$[0.0172, 0.0927]$ (s^{-1})
$k3$	Dephosphorylation Rate of IRAK4p*	$[0.0128, 0.0993]$ (s^{-1})
$k4f$	Association between IRAK4p* + IRAK1	$[9.9\text{x}10^{-3}, 0.0977]$ ($nM^{-1}s^{-1}$)
$k4r$	Dissociation of IRAK4p*-IRAK1	$[4.3\text{x}10^{-3}, 0.0982]$ (s^{-1})
$k5$	Dephosphorylation Rate of IRAK1p*	$[4.7\text{x}10^{-3}, 0.0988]$ (s^{-1})
$k6f$	Association between IRAK1p* + TRAF6	$[9.7\text{x}10^{-3}, 0.0680]$ ($nM^{-1}s^{-1}$)
$k6r$	Dissociation of IRAK1p*-TRAF6	$[0.0172, 0.0640]$ (s^{-1})
$k7f$	Association between IRAK1p*-TRAF6 + TABTAK	$[3.4\text{x}10^{-3}, 0.0653]$ ($nM^{-1}s^{-1}$)
$k7r$	Dissociation of IRAK1p*-TRAF6-TABTAK	$[0.0280, 0.0987]$ (s^{-1})
$k8$	Dephosphorylation Rate of TABTAKp*	$[3.\text{x}10^{-3}, 0.4331]$ (s^{-1})
$k9f$	Association between TABTAKp* + MKK4/7	$[1.4\text{x}10^{-3}, 0.0753]$ ($nM^{-1}s^{-1}$)
$k9r$	Dissociation of TABTAKp*-MKK4/7	$[1.6\text{x}10^{-3}, 0.0732]$ (s^{-1})
$k10$	Dephosphorylation Rate of MKK4/7p*	$[2.1\text{x}10^{-3}, 0.0683]$ (s^{-1})
$k11f$	Association between MKK4/7p* + JNK	$[4.5\text{x}10^{-3}, 0.4935]$ ($nM^{-1}s^{-1}$)
$k11r$	Dissociation of MKK4/7p*-JNK	$[8.9\text{x}10^{-3}, 0.0906]$ (s^{-1})
$k12$	Dephosphorylation Rate of JNKp*	$[3.3\text{x}10^{-3}, 0.0789]$ (s^{-1})
$k13f$	Import Rate to Nucleus of JNKp*	$[0.0242, 0.0854]$ ($nM^{-1}s^{-1}$)
$k13r$	Export Rate from Nucleus of JNKp*n	$[2.6\text{x}10^{-3}, 0.0751]$ (s^{-1})
$k14f$	Association between TABTAKp* + MKK3/6	$[4.8\text{x}10^{-3}, 0.0809]$ ($nM^{-1}s^{-1}$)
$k14r$	Dissociation of TABTAKp*-MKK3/6	$[0.0219, 0.0987]$ (s^{-1})
$k15$	Dephosphorylation Rate of MKK3/6p*	$[0.0111, 0.0792]$ (s^{-1})
$k16f$	Association between MKK3/6p* + p38	$[3.9\text{x}10^{-3}, 0.0902]$ ($nM^{-1}s^{-1}$)
$k16r$	Dissociation of MKK3/6p*-p38	$[0.0125, 0.1788]$ (s^{-1})
$k17$	Dephosphorylation Rate of p38p*	$[8.2\text{x}10^{-3}, 0.4369]$ (s^{-1})
$k18f$	Import Rate to Nucleus of p38p*	$[0.0163, 0.0876]$ ($nM^{-1}s^{-1}$)
$k18r$	Export Rate from Nucleus of p38p*n	$[6.6\text{x}10^{-3}, 0.6447]$ (s^{-1})
$k19f$	Association between TABTAKp* + IKKc	$[0.0374, 1.3332]$ ($nM^{-1}s^{-1}$)
$k19r$	Dissociation of TABTAKp*-IKKc	$[5.9\text{x}10^{-3}, 0.0974]$ (s^{-1})
$k20$	Dephosphorylation Rate of IKKcp*	$[0.0313, 0.0990]$ (s^{-1})
$k21f$	Association between IKKcp* + IκB-NFκB	$[0.0199, 0.3221]$ ($nM^{-1}s^{-1}$)
$k21r$	Dissociation of IKKcp*-IκB-NFκB	$[4.8\text{x}10^{-3}, 0.0516]$ (s^{-1})
$k22f$	Import Rate to Nucleus of NFκB	$[3.6\text{x}10^{-3}, 0.5451]$ ($nM^{-1}s^{-1}$)
$k22r$	Export Rate from Nucleus of NFκBn	$[0.0265, 0.0763]$ (s^{-1})
$k23f$	Association between IKKcp* + TpL2	$[9.6\text{x}10^{-3}, 0.0953]$ ($nM^{-1}s^{-1}$)
$k23r$	Dissociation of IKKcp*-TpL2	$[0.0291, 0.3354]$ (s^{-1})
$k24$	Dephosphorylation Rate of TpL2p*	$[5.0\text{x}10^{-3}, 0.0872]$ (s^{-1})
$k25f$	Association between TpL2p* + MKK1/2	$[6.4\text{x}10^{-3}, 1.7256]$ ($nM^{-1}s^{-1}$)
$k25r$	Dissociation of TpL2p*-MKK1/2	$[0.0135, 0.7698]$ (s^{-1})
$k26$	Dephosphorylation Rate of MKK1/2p*	$[2.0\text{x}10^{-3}, 0.0970]$ (s^{-1})
$k27f$	Association between MKK1/2p* + ERK	$[0.0234, 0.0870]$ ($nM^{-1}s^{-1}$)
$k27r$	Dissociation of MKK1/2p*-ERK	$[3.5\text{x}10^{-3}, 0.1723]$ (s^{-1})
$k28$	Dephosphorylation Rate of ERKp*	$[0.0195, 0.0814]$ (s^{-1})
$k29f$	Import Rate to Nucleus of ERKp*	$[0.01001, 0.7902]$ ($nM^{-1}s^{-1}$)
$k29r$	Export Rate from Nucleus of ERKp*n	$[0.0107, 0.0987]$ (s^{-1})
$k30f$	Association between TLR4 + I_1	$[9.4\text{x}10^{-3}, 0.0689]$ ($nM^{-1}s^{-1}$)
$k30r$	Dissociation of TLR4-I_1	$[0.0109, 2.1297]$ (s^{-1})
$k31f$	Association between TLR4-I_1 + I_2	$[0.0119, 0.08026]$ ($nM^{-1}s^{-1}$)
$k31r$	Dissociation of TLR4-I_1-I_2	$[0.0281, 1.0450]$ (s^{-1})

Supplementary Table 4 - Reaction parameters, and their corresponding biochemical properties, used for simulating the ensemble of 10 predictive dynamical trajectories.

Reaction Parameter	Biochemical Property	Range
$k32f$	Association between TLR4-I_1-I_2 + I_3	$[2.2\text{x}10^{-3}, 0.0898]$ ($nM^{-1}s^{-1}$)
$k32r$	Dissociation of TLR4-I_1-I_2-I_3	$[0.0103, 0.0775]$ (s^{-1})
$k33f$	Association between TLR4-I_1-I_2-I_3 + TRAM	$[0.0194, 0.0991]$ ($nM^{-1}s^{-1}$)
$k33r$	Dissociation of TLR4-I_1-I_2-I_3-TRAM	$[0.0417, 0.1498]$ (s^{-1})
$k34f$	Association between TLR4-I_1-I_2-I_3-TRAM + TRIF	$[5.2\text{x}10^{-3}, 0.2594]$ ($nM^{-1}s^{-1}$)
$k34r$	Dissociation of TLR4-I_1-I_2-I_3-TRAM-TRIF	$[0.0157, 0.5049]$ (s^{-1})
$k35f$	Association between TLR4-I_1-I_2-I_3-TRAM-TRIF + RIP1	$[5.5\text{x}10^{-3}, 0.1454]$ ($nM^{-1}s^{-1}$)
$k35r$	Dissociation of TLR4-I_1-I_2-I_3-TRAM-TRIF-RIP1	$[0.0232, 0.0957]$ (s^{-1})
$k36f$	Association between TLR4-I_1-I_2-I_3-TRAM-TRIF-RIP1 + AIP1	$[0.0198, 0.6388]$ ($nM^{-1}s^{-1}$)
$k36r$	Dissociation of TLR4-I_1-I_2-I_3-TRAM-TRIF-RIP1-AIP1	$[0.0266, 0.0753]$ (s^{-1})
$k37f$	Association between TLR4-I_1-I_2-I_3-TRAM-TRIF-RIP1-AIP1 + TRAF6	$[5.0\text{x}10^{-3}, 0.1734]$ ($nM^{-1}s^{-1}$)
$k37r$	Dissociation of TLR4-I_1-I_2-I_3-TRAM-TRIF-RIP1-AIP1-TRAF6	$[6.3\text{x}10^{-3}, 0.1642]$ (s^{-1})
$k38f$	Association between TLR4-I_1-I_2-I_3-TRAM-TRIF + TRAF6	$[0.0417, 0.0950]$ ($nM^{-1}s^{-1}$)
$k38r$	Dissociation of TLR4-I_1-I_2-I_3-TRAM-TRIF-TRAF6	$[5.0\text{x}10^{-4}, 0.0946]$ (s^{-1})
$k39f$	Association between TLR4-I_1-I_2-I_3-TRAM-TRIF + TBK1	$[0.0154, 0.0939]$ ($nM^{-1}s^{-1}$)
$k39r$	Dissociation of TLR4-I_1-I_2-I_3-TRAM-TRIF-TBK1	$[0.0152, 1.8878]$ (s^{-1})
$k40f$	Association between TLR4-I_1-I_2-I_3-TRAM-TRIF-TBK1 + IRF	$[1.8\text{x}10^{-3}, 0.0641]$ ($nM^{-1}s^{-1}$)
$k40r$	Dissociation of TLR4-I_1-I_2-I_3-TRAM-TRIF-TBK1-IRF	$[9.2\text{x}10^{-3}, 0.1056]$ (s^{-1})
$k41$	Dephosphorylation Rate of IRFp*	$[0.0304, 1.2562]$ (s^{-1})
$k42f$	Dimerization of IRFp* with IRFp*	$[0.0142, 0.2026]$ ($nM^{-1}s^{-1}$)
$k42r$	Dissociation of IRFpp*	$[0.0136, 1.3562]$ (s^{-1})
$k43f$	Export Rate to Nucleus of IRFpp*	$[9.4\text{x}10^{-3}, 0.0791]$ ($nM^{-1}s^{-1}$)
$k43r$	Import Rate from Nucleus of IRFpp*n	$[4.4\text{x}10^{-3}, 0.0816]$ (s^{-1})
$k44f$	Association between JNKp*n and AP1n	$[2.6\text{x}10^{-4}, 0.0939]$ ($nM^{-1}s^{-1}$)
$k44r$	Dissociation of JNKp*n-AP1n	$[8.1\text{x}10^{-3}, 0.0525]$ (s^{-1})
$k45f$	Association between p38p*n and AP1n	$[9.9\text{x}10^{-3}, 0.1876]$ ($nM^{-1}s^{-1}$)
$k45r$	Dissociation of p38p*n-AP1n	$[2.5\text{x}10^{-3}, 0.1170]$ (s^{-1})
$k46f$	Association between ERKp*n and AP1n	$[4.5\text{x}10^{-3}, 0.0821]$ ($nM^{-1}s^{-1}$)
$k46r$	Dissociation of ERKp*n-AP1n	$[2.8\text{x}10^{-3}, 0.0948]$ (s^{-1})
$k2cat$	Phosphorylation Rate of IRAK4	$[9.9\text{x}10^{-4}, 0.0957]$ (s^{-1})
$k4cat$	Phosphorylation Rate of IRAK1	$[5.8\text{x}10^{-4}, 0.9318]$ (s^{-1})
$k7cat$	Phosphorylation Rate of TABTAK	$[6.2\text{x}10^{-3}, 0.1477]$ (s^{-1})
$k9cat$	Phosphorylation Rate of MKK4/7	$[0.0245, 0.3642]$ (s^{-1})
$k11cat$	Phosphorylation Rate of JNK	$[3.3\text{x}10^{-3}, 0.0643]$ (s^{-1})
$k14cat$	Phosphorylation Rate of MKK3/6	$[8.3\text{x}10^{-3}, 0.0950]$ (s^{-1})
$k16cat$	Phosphorylation Rate of p38	$[0.0300, 0.0927]$ (s^{-1})
$k19cat$	Phosphorylation Rate of IKKc	$[0.0961, 1.5261]$ (s^{-1})
$k21cat$	Dissociation Rate of IκB-NFκB	$[2.4\text{x}10^{-3}, 0.0956]$ (s^{-1})
$k23cat$	Phosphorylation Rate of TpL2	$[9.2\text{x}10^{-4}, 0.0878]$ (s^{-1})
$k25cat$	Phosphorylation Rate of MKK1/2	$[3.7\text{x}10^{-3}, 0.0514]$ (s^{-1})
$k27cat$	Phosphorylation Rate of ERK	$[1.9\text{x}10^{-3}, 0.1177]$ (s^{-1})
$k40cat$	Phosphorylation Rate of IRF	$[0.6631, 0.9876]$ (s^{-1})
$\alpha 1$	Transcriptional Strength of AP1 over *Tnfα*	$[0.4139, 3.7742]$
$\beta 1$	Transcriptional Strength of NFκB over *Tnfα*	$[0.0691, 3.8909]$
$VA1$	Cooperativity Effects of AP1 on *Tnfα* Transcription	$[6.0094, 9.9702]$
$VB1$	Cooperativity Effects of NFκB on *Tnfα* Transcription	$[4.0045, 6.7778]$
$K\beta 1$	MichaelisMenten-Constant Related to *Tnfα* Transcription	$[4.5178, 22.3941]$ (nM)
k_dTnf	Degradation Rate of *Tnfα* mRNA	$[0.0140, 0.0992]$ (h^{-1})
$\alpha 2$	Transcriptional Strength of IRFpp* over *Cxcl10*	$[0.5774, 1.9506]$
$\beta 2$	Transcriptional Strength of NFκB over *Cxcl10*	$[0.6439, 1.9872]$
$VA2$	Cooperativity Effects of IRFpp* on *Cxcl10*	$[4.0094, 7.2702]$
$VB2$	Cooperativity Effects of NFκB on *Cxcl10*	$[3.1194, 5.1711]$
$K\beta 2$	MichaelisMenten-Constant Related to *Cxcl10* Transcription	$[0.7288, 22.5447]$ (nM)
k_dCxc	Degradation Rate of *Cxcl10* mRNA	$[7.7\text{x}10^{-4}, 0.0979]$ (h^{-1})
$TmaxTnf$	Max. Transcriptional Rate of *Tnfα*	$[0.8637, 23.8489]$ (nM h^{-1})
$TmaxCxc$	Max. Transcriptional Rate of *Cxcl10*	$[0.7310, 13.4748]$ (nM h^{-1})
ρTnf	Transcriptional Efficiency of the *Tnfα* Promoter	$[0.0231, 0.2059]$
ρCxc	Transcriptional Efficiency of the *Cxcl10* Promoter	$[0.0102, 0.1111]$

Initial conditions

Supplementary Table 5 - Initial concentrations assigned to each reaction species modeled.

Reaction Species	Exp 1 Range (nm)	Exp 2 (nm)	Reaction Species	Exp 1 Range (nm)	Exp 2 (nm)
TLR4s	[0.055, 0.162]	0.111	IKKcp*TpL2	[0.000, 0.000]	0.000
TLR4a	[0.199, 0.587]	0.393	TpL2p*	[0.000, 0.000]	0.000
MyD88Mal	[0.510, 1.463]	0.987	MKK12	[0.516, 1.465]	0.979
MyD88MalTLR4a	[0.375, 1.066]	0.734	TpL2p*MKK12	[0.000, 0.000]	0.000
MyD88MalTLR4aIRAK4	[0.271, 0.795]	0.535	MKK12p*	[0.000, 0.000]	0.000
IRAK4	[0.161, 0.481]	0.321	ERK	[0.120, 0.354]	0.237
IRAK4p*	[0.473, 1.391]	0.931	MKK12p*ERK	[0.302, 0.892]	0.595
IRAK4pIRAK1	[0.000, 0.000]	0.000	ERKp*	[0.140, 0.416]	0.278
IRAK1	[0.009, 0.028]	0.019	ERKp*n	[0.000, 0.000]	0.000
IRAK1p*	[0.042, 0.124]	0.083	Intermediary-1(I1)	[0.472, 1.361]	0.925
TRAF6	[0.335, 0.999]	0.668	TLR4aI1	[0.000, 0.000]	0.000
IRAK1p*TRAF6	[0.068, 0.199]	0.134	Intermediary-2 (I2)	[0.383, 1.129]	0.753
TABTAK	[0.421, 1.247]	0.841	TLR4aI1I2	[0.000, 0.000]	0.000
IRAK1p*TRAF6TABTAK	[0.000, 0.000]	0.000	Intermediary-3 (I3)	[0.029, 0.084]	0.057
TABTAKp*	[0.000, 0.000]	0.000	TLR4aI1I2I3	[0.179, 0.513]	0.344
MKK47	[0.092, 0.273]	0.182	TRAM	[0.493, 1.446]	0.972
TABTAKp*MKK47	[0.000, 0.000]	0.000	TLR4aI1I2I3TRAM	[0.000, 0.000]	0.000
MKK47p*	[0.490, 1.464]	0.976	TRIF	[0.484, 1.418]	0.949
JNK	[0.353, 1.050]	0.702	TLR4aI1I2I3TRAMTRIF	[0.389, 1.166]	0.779
MKK47p*JNK	[0.000, 0.000]	0.000	RIP1	[0.453, 1.288]	0.871
JNKp*	[0.225, 0.669]	0.446	TLR4aI1I2I3TRAMTRIFRIP1	[0.000, 0.000]	0.000
JNKp*n	[0.000, 0.000]	0.000	AIP1	[0.266, 0.777]	0.519
MKK36	[0.272, 0.801]	0.128	TLR4aI1I2I3TRAMTRIFRIP1AIP1	[0.000, 0.000]	0.000
TABTAKp*MKK36	[0.288, 0.812]	0.543	TLR4aI1I2I3TRAMTRIFRIP1AIP1TRAF6	[0.000, 0.000]	0.000
MKK36p*	[0.007, 0.019]	0.013	TLR4aI1I2I3TRAMTRIFTRAF6	[0.000, 0.000]	0.000
P38	[0.137, 0.397]	0.265	TLR4aI1I2I3TRAMTRIFTBK1	[0.000, 0.000]	0.000
MKK36p*P38	[0.109, 0.323]	0.216	TBK1	[0.410, 1.197]	0.807
P38p*	[0.000, 0.000]	0.000	IRF	[0.355, 1.015]	0.696
P38p*n	[0.000, 0.000]	0.000	TLR4aI1I2I3TRAMTRIFTBK1IRF	[0.000, 0.000]	0.000
IKKc	[0.432, 1.277]	0.851	IRFp*	[0.210, 0.626]	0.420
TABTAKp*IKKc	[0.000, 0.000]	0.000	IRF2p*	[0.475, 1.396]	0.936
IKKcp*	[0.000, 0.000]	0.000	IRF2p*n	[0.000, 0.000]	0.000
IkBNFkB	[0.427, 1.269]	0.847	AP1	[0.000, 0.000]	0.000
IKKcp*IkBNFkB	[0.264, 0.771]	0.519	JNKp*nAP1	[0.157, 0.452]	0.303
IkB	[0.000, 0.000]	0.000	P38p*nAP1	[0.000, 0.000]	0.000
NFkB	[0.000, 0.000]	0.000	ERKp*nAP1	[0.416, 1.237]	0.825
NFkBn	[0.000, 0.000]	0.000	*Tnf*-alpha	[0.000, 0.000]	0.000
TpL2	[0.164, 0.461]	0.313	*Cxcl10*	[0.000, 0.000]	0.000

Rate equations

An exponential decay temporal profile for the LPS (ligand) concentration was implemented in this way:

$$LPS(t) = Exp^{-0.01*t}$$

The rate equations shown below were constructed on the basis of mass-balance principles. It is also worth noting that signaling fluxes involve transactions of information in terms of biochemical reactions, as opposed to those mass and energy fluxes sustaining metabolic processes.

1) Temporal variation in the concentration of Rs (the TLR4 susceptible form):

$$\frac{d[Rs]}{dt} = k_{ps} - k_{s\mapsto a}[Rs]\left[\frac{[LPS]^n}{K_b^n + [LPS]^n}\right] + k_{a\mapsto s}[Ra] - k_{ds}[Rs]$$

2) Temporal variation in the concentration of Ra (the TLR4 activated form):

$$\frac{d[Ra]}{dt} = k_{s\mapsto a}[Rs]\left[\frac{[LPS]^n}{K_b^n + [LPS]^n}\right] - k_{a\mapsto s}[Ra] - k_{da}[Ra]$$

3) Temporal variation in the concentration of the reaction species MyD88/Mal:

$$\frac{d[MyD88/Mal]}{dt} = -(k_{1f}[MyD88/Mal][Ra] - k_{1r}[MyD88/Mal - Ra])$$

4) Temporal variation in the concentration of the complex MyD88/Mal-Ra:

$$\begin{aligned}\frac{d[MyD88/Mal - Ra]}{dt} &= \\ &(k_{1f}[MyD88/Mal][Ra] - k_{1r}[MyD88/Mal - Ra]) - \\ &(k_{2f}[MyD88/Mal - Ra][IRAK4] - k_{2r}[MyD88/Mal - Ra - IRAK4]) + \\ &(k_{2cat}[MyD88/Mal - Ra - IRAK4])\end{aligned}$$

5) Temporal variation in the concentration of the complex MyD88/Mal-Ra-IRAK4:

$$\begin{aligned}\frac{d[MyD88/Mal - Ra - IRAK4]}{dt} &= \\ &(k_{2f}[MyD88/Mal - Ra][IRAK4] - k_{2r}[MyD88/Mal - Ra - IRAK4]) - \\ &(k_{2cat}[MyD88/Mal - Ra - IRAK4])\end{aligned}$$

6) Temporal variation in the concentration of the reaction species IRAK4:

$$\begin{aligned}\frac{d[IRAK4]}{dt} &= \\ &- (k_{2f}[MyD88/Mal - Ra][IRAK4] - k_{2r}[MyD88/Mal - Ra - IRAK4]) + (k_3 IRAK4p^*)\end{aligned}$$

7) Temporal variation in the concentration of the reaction species IRAK4p*:

$$\begin{aligned}\frac{d[IRAK4p^*]}{dt} &= \\ &- (k_3 IRAK4p^*) + (k_{2cat}[MyD88/Mal - Ra - IRAK4]) - \\ &(k_{4f}[IRAK4p^*][IRAK1] - k_{4r}[IRAK4p^* - IRAK1]) + \\ &(k_{4cat}[IRAK4p^* - IRAK1])\end{aligned}$$

8) Temporal variation in the concentration of the complex IRAK4p*-IRAK1:

$$\frac{d[IRAK4p^* - IRAK1]}{dt} = (k_{4f}[IRAK4p^*][IRAK1] - k_{4r}[IRAK4p^* - IRAK1]) - (k_{2cat}[MyD88/Mal - Ra - IRAK4])$$

9) Temporal variation in the concentration of the reaction species IRAK1:

$$\frac{d[IRAK1]}{dt} = -(k_{4f}[IRAK4p^*][IRAK1] - k_{4r}[IRAK4p^* - IRAK1]) + (k_5[IRAK1p^*])$$

10) Temporal variation in the concentration of the reaction species IRAK1p*:

$$\frac{d[IRAK1p^*]}{dt} = -(k_5[IRAK1p^*]) + (k_{4cat}[IRAK4p^* - IRAK1]) - (k_{6f}[IRAK1p^*] - k6r[IRAK1p^* - TRAF6])$$

11) Temporal variation in the concentration of the reaction species TRAF6:

$$\frac{d[TRAF6]}{dt} = -(k_{6f}[IRAK1p^*] - k6r[IRAK1p^* - TRAF6]) - (k_{37f}[RaI_1I_2I_3 - TRIF - TRAM - RIP - AIP1][TRAF6] - k_{37r}[RaI_1I_2I_3 - TRIF - TRAM - RIP - AIP1 - TRAF6]) - (k_{38f}[RaI_1I_2I_3 - TRIF - TRAM][TRAF6] - k_{38r}[RaI_1I_2I_3 - TRIF - TRAM - TRAF6])$$

12) Temporal variation in the concentration of the complex IRAK1p*-TRAF6:

$$\frac{d[IRAK1p^* - TRAF6]}{dt} = -(k_{6f}[IRAK1p^*] - k6r[IRAK1p^* - TRAF6]) - + (k_{7f}[IRAK1p^* - TRAF6][TABTAK] - k_{7r}[IRAK1p^* - TRAF6 - TABTAK]) + (k_{7cat}[IRAK1p^* - TRAF6 - TABTAK])$$

13) Temporal variation in the concentration of the reaction species TABTAK:

$$\frac{d[TABTAK]}{dt} = -(k_{7f}[IRAK1p^* - TRAF6][TABTAK] - k_{7r}[IRAK1p^* - TRAF6 - TABTAK]) + (k_8[TABTAKp^*])$$

14) Temporal variation in the concentration of the complex IRAK1p*-TRAF6-TABTAK:

$$\frac{d[IRAK1p^* - TRAF6 - TABTAK]}{dt} = (k_{7f}[IRAK1p^* - TRAF6][TABTAK] - k_{7r}[IRAK1p^* - TRAF6 - TABTAK]) - (k_{7cat}[IRAK1p^* - TRAF6 - TABTAK])$$

15) Temporal variation in the concentration of the reaction species TABTAKp*:

$$\frac{d[TABTAKp^*]}{dt} = (k_{7cat}[IRAK1p^* - TRAF6 - TABTAK]) - (k_8[TABTAKp^*]) - (k_{9f}[TABTAKp^*][MKK4/7] - k_{9r}[TABTAKp^* - MKK4/7]) + (k_{9cat}[TABTAKp^* - MKK4/7])$$

16) Temporal variation in the concentration of the reaction species MKK4/7:

$$\frac{d[MKK4/7]}{dt} = -(k_{9f}[TABTAKp^*][MKK4/7] - k_{9r}[TABTAKp^* - MKK4/7]) + (k_{10}[MKK4/7p^*])$$

17) Temporal variation in the concentration of the complex TABTAKp*-MKK4/7:

$$\frac{d[TABTAKp^* - MKK4/7]}{dt} = (k_{9f}[TABTAKp^*][MKK4/7] - k_{9r}[TABTAKp^* - MKK4/7]) - (k_{9cat}[TABTAKp^* - MKK4/7])$$

18) Temporal variation in the concentration of the reaction species MKK4/7p*:

$$\frac{d[MKK4/7p^*]}{dt} = (k_{9cat}[TABTAKp^* - MKK4/7]) - (k_{10}[MKK4/7p^*]) - (k_{11f}[MKK4/7p^*][JNK] - k_{11r}[MKK4/7p^* - JNK]) + (k_{11cat}[MKK4/7p^* - JNK])$$

19) Temporal variation in the concentration of the reaction species JNK:

$$\frac{d[JNK]}{dt} = -(k_{11f}[MKK4/7p^*][JNK] - k_{11r}[MKK4/7p^* - JNK]) + (k_{12}[JNKp^*])$$

20) Temporal variation in the concentration of the complex MKK4/7p*-JNK:

$$\frac{d[MKK4/7p^* - JNK]}{dt} = (k_{11f}[MKK4/7p^*][JNK] - k_{11r}[MKK4/7p^* - JNK]) + (k_{11cat}[MKK4/7p^* - JNK])$$

21) Temporal variation in the concentration of the reaction species JNKp*:

$$\frac{d[JNKp^*]}{dt} = (k_{11cat}[MKK4/7p^* - JNK]) - (k_{12}[JNKp^*]) - (k_{13f}[JNKp^*] - k_{13r}[JNKp^*n])$$

22) Temporal variation in the concentration of the reaction species JNKp*n:

$$\frac{d[JNKp^*n]}{dt} = (k_{13f}[JNKp^*] - k_{13r}[JNKp^*n]) - (k_{44f}[JNKp^*n][AP1n] - k_{44r}[JNKp^*n - AP1n])$$

23) Temporal variation in the concentration of the reaction species MKK3/6:

$$\frac{d[MKK3/6]}{dt} = -(k_{14f}[TABTAKp^*][MKK3/6] - k_{14r}[TABTAKp^* - MKK3/6]) + (k_{15}[MKK3/6p^*])$$

24) Temporal variation in the concentration of the complex TABTAKp*-MKK3/6:

$$\frac{d[TABTAKp^* - MKK3/6]}{dt} = (k_{14f}[TABTAKp^*][MKK3/6] - k_{14r}[TABTAKp^* - MKK3/6]) - (k_{14cat}[TABTAKp^* - MKK3/6])$$

25) Temporal variation in the concentration of the reaction species MKK3/6-p*:

$$\frac{d[MKK3/6p^*]}{dt} = (k_{14cat}[TABTAKp^* - MKK3/6]) - (k_{15}[MKK3/6p^*]) - (k_{16f}[MKK3/6p^*][P38] - k_{16r}[MKK3/6p^* - P38]) + (k_{16cat}[MKK3/6p^* - P38])$$

26) Temporal variation in the concentration of the reaction species P38:

$$\frac{d[MKK3/6p^*]}{dt} = -(k_{16f}[MKK3/6p^*][P38] - k_{16r}[MKK3/6p^* - P38]) + (k_{17}[P38p^*])$$

27) Temporal variation in the concentration of the complex MKK3/6p*-P38:

$$\frac{d[MKK3/6p^* - P38]}{dt} = (k_{16f}[MKK3/6p^*][P38] - k_{16r}[MKK3/6p^* - P38]) - (k_{16cat}[MKK3/6p^* - P38])$$

28) Temporal variation in the concentration of the reaction species P38p*:

$$\frac{d[P38p^*]}{dt} = (k_{16cat}[MKK3/6p^* - P38]) - (k_{17}[P38p^*]) - (k_{18f}[P38p^*] - k_{18r}[P38p^*n])$$

29) Temporal variation in the concentration of the reaction species P38p*n:

$$\frac{d[P38p^*n]}{dt} = (k_{18f}[P38p^*] - k_{18r}[P38p^*n]) - (k_{45f}[P38p^*n][AP1n] - k_{45r}[P38p^*n - AP1n])$$

30) Temporal variation in the concentration of the reaction species IKKc:

$$\frac{d[IKKc]}{dt} = -(k_{19f}[TABTAKp^*][IKKc] - k_{19r}[TABTAKp^* - IKKc]) + (k_{20}[IKKcp^*])$$

31) Temporal variation in the concentration of the complex TABTAKp*-IKKc:

$$\frac{d[TABTAKp^* - IKKc]}{dt} =$$
$$(k_{19f}[TABTAKp^*][IKKc] - k_{19r}[TABTAKp^* - IKKc]) - (k_{19cat}[TABTAKp^* - IKKc])$$

32) Temporal variation in the concentration of the reaction species IKKcp*:

$$\frac{d[IKKcp^*]}{dt} =$$
$$(k_{19cat}[TABTAKp^* - IKKc]) - (k_{20}[IKKcp^*]) -$$
$$(k_{21f}[IKKcp^*][I\kappa B - NF\kappa B] - k_{21r}[IKKcp^* - I\kappa B - NF\kappa B]) + (k_{21cat}[IKKcp^* - I\kappa B - NF\kappa B]) -$$
$$(k_{23f}[IKKcp^*][TpL2] - k_{23r}[IKKcp^*TpL2]) + (k_{23cat}[IKKcp^*TpL2])$$

33) Temporal variation in the concentration of the complex IκB-NFκB:

$$\frac{d[I\kappa B - NF\kappa B]}{dt} =$$
$$-(k_{21f}[IKKcp^*][I\kappa B - NF\kappa B] - k_{21r}[IKKcp^* - I\kappa B - NF\kappa B])$$

34) Temporal variation in the concentration of the complex IKKcp*-IκB-NFκB:

$$\frac{d[IKKcp^* - I\kappa B - NF\kappa B]}{dt} =$$
$$(k_{21f}[IKKcp^*][I\kappa B - NF\kappa B] - k_{21r}[IKKcp^* - I\kappa B - NF\kappa B])$$

35) Temporal variation in the concentration of the reaction species IκB:

$$\frac{d[I\kappa B]}{dt} = (k_{21cat}[IKKcp^* - I\kappa B - NF\kappa B])$$

36) Temporal variation in the concentration of the reaction species NFκB:

$$\frac{d[NF\kappa B]}{dt} = (k_{21cat}[IKKcp^* - I\kappa B - NF\kappa B]) - (k_{22f}[NF\kappa B] - k_{22r}[NF\kappa Bn])$$

37) Temporal variation in the concentration of the reaction species NFκBn:

$$\frac{d[NF\kappa Bn]}{dt} = (k_{22f}[NF\kappa B] - k_{22r}[NF\kappa Bn])$$

38) Temporal variation in the concentration of the reaction species TpL2:

$$\frac{d[TpL2]}{dt} = -(k_{23f}[IKKcp^*][TpL2] - k_{23r}[IKKcp^*TpL2]) + (k_{24}[TpL2*])$$

39) Temporal variation in the concentration of the complex IKKcp*-TpL2:

$$\frac{d[IKKcp^* - TpL2]}{dt} = (k_{23f}[IKKcp^*][TpL2] - k_{23r}[IKKcp^*TpL2]) + (k_{24}[TpL2p*]) - (k_{23cat}[IKKcp^*TpL2])$$

40) Temporal variation in the concentration of the reaction species TpL2p*:

$$\frac{d[TpL2p^*]}{dt} = (k_{23cat}[IKKcp^*TpL2]) - (k_{24}[TpL2p*]) - (k_{25f}[TpL2p*][MKK1/2] - k_{25r}[TpL2p* - MKK1/2]) - (k_{25cat}[TpL2p* - MKK1/2])$$

41) Temporal variation in the concentration of the reaction species MKK1/2:

$$\frac{d[MKK1/2]}{dt} = -(k_{25f}[TpL2p*][MKK1/2] - k_{25r}[TpL2p* - MKK1/2]) + (k_{26}[MKK1/2p*])$$

42) Temporal variation in the concentration of the complex TpL2p*-MKK1/2:

$$\frac{d[TpL2p^* - MKK1/2]}{dt} = (k_{25f}[TpL2p*][MKK1/2] - k_{25r}[TpL2p* - MKK1/2]) - (k_{25cat}[TpL2p* - MKK1/2])$$

43) Temporal variation in the concentration of the reaction species MKK1/2p*:

$$\frac{d[MKK1/2p^*]}{dt} = (k_{25cat}[TpL2p* - MKK1/2]) - (k_{26}[MKK1/2p*]) - (k_{27f}[MKK1/2p*][ERK] - k_{27r}[MKK1/2p*ERK]) + (k_{27cat}[MKK1/2p*ERK])$$

44) Temporal variation in the concentration of the reaction species ERK:

$$\frac{d[ERK]}{dt} = -(k_{27f}[MKK1/2p*][ERK] - k_{27r}[MKK1/2p*ERK]) + (k_{28}[ERKp*])$$

45) Temporal variation in the concentration of the complex MKK1/2p*-ERK:

$$\frac{d[MKK1/2p^* - ERK]}{dt} = (k_{27f}[MKK1/2p*][ERK] - k_{27r}[MKK1/2p*ERK]) - (k_{27cat}[MKK1/2p*ERK])$$

46) Temporal variation in the concentration of the reaction species ERKp*:

$$\frac{d[ERKp^*]}{dt} = (k_{27cat}[MKK1/2p*ERK]) - (k_{28}[ERKp*]) - (k_{29f}[ERKp*] - k_{29r}[ERKp*n])$$

47) Temporal variation in the concentration of the reaction species ERKp*n:

$$\frac{d[ERKp^*n]}{dt} = (k_{29f}[ERKp*] - k_{29r}[ERKp*n]) - (k_{46f}[ERKp*n][AP1n] - k_{46r}[ERKp*n - AP1n])$$

48) Temporal variation in the concentration of the reaction species I1 (hypothetical intermediary/adapter molecule 1):

$$\frac{d[I1]}{dt} = -(k_{30f}[Ra][I1] - k_{30r}[RaI1])$$

49) Temporal variation in the concentration of the complex Ra-I1:

$$\frac{d[RaI1]}{dt} = (k_{30f}[Ra][I1] - k_{30r}[RaI1])$$

50) Temporal variation in the concentration of the reaction species I2 (hypothetical intermediary/adapter molecule 2):

$$\frac{d[I2]}{dt} = -(k_{31f}[RaI1][I2] - k_{31r}[RaI1I2])$$

51) Temporal variation in the concentration of the complex Ra-I1-I2:

$$\frac{d[RaI1I2]}{dt} = (k_{31f}[RaI1][I2] - k_{31r}[RaI1I2])$$

52) Temporal variation in the concentration of the reaction species I3 (hypothetical intermediary/adapter molecule 3):

$$\frac{d[I3]}{dt} = -(k_{32f}[RaI1I2][I3] - k_{32r}[RaI1I2I3])$$

53) Temporal variation in the concentration of the complex Ra-I1-I2-I3:

$$\frac{d[RaI1I2I3]}{dt} = (k_{32f}[RaI1I2][I3] - k_{32r}[RaI1I2I3])$$

54) Temporal variation in the concentration of the reaction species TRAM:

$$\frac{d[RaI1I2I3]}{dt} = -(k_{33f}[RaI1I2I3][TRAM] - k_{33r}[RaI1I2I3T])$$

55) Temporal variation in the concentration of the complex Ra-I1-I2-I3-TRAM:

$$\frac{d[RaI1I2I3T]}{dt} = (k_{33f}[RaI1I2I3][TRAM] - k_{33r}[RaI1I2I3T])$$

56) Temporal variation in the concentration of the reaction species TRIF:

$$\frac{d[TRIF]}{dt} = -(k_{34f}[RaI1I2I3T][TRIF] - k_{34r}[RaI1I2I3TT])$$

57) Temporal variation in the concentration of the complex Ra-I1-I2-I3-TRAM-TRIF:

$$\frac{d[RaI1I2I3TT]}{dt} = (k_{34f}[RaI1I2I3T][TRIF] - k_{34r}[RaI1I2I3TT])$$

58) Temporal variation in the concentration of the reaction species RIP1:

$$\frac{d[RIP1]}{dt} = -(k_{35f}[RaI1I2I3TT][RIP1] - k_{35r}[RaI1I2I3TTR])$$

59) Temporal variation in the concentration of the complex Ra-I1-I2-I3-TRAM-TRIF-RIP1:

$$\frac{d[RaI1I2I3TTR]}{dt} = (k_{35f}[RaI1I2I3TT][RIP1] - k_{35r}[RaI1I2I3TTR])$$

60) Temporal variation in the concentration of the reaction species AIP1:

$$\frac{d[AIP1]}{dt} = -(k_{36f}[RaI1I2I3TTR][AIP1] - k_{36r}[RaI1I2I3TTRA])$$

61) Temporal variation in the concentration of the complex Ra-I1-I2-I3-TRAM-TRIF-RIP1-AIP1:

$$\frac{d[RaI1I2I3TTRA]}{dt} = (k_{36f}[RaI1I2I3TTR][AIP1] - k_{36r}[RaI1I2I3TTRA])$$

62) Temporal variation in the concentration of the complex Ra-I1-I2-I3-TRAM-TRIF-RIP1-AIP1-TRAF6:

$$\frac{d[RaI1I2I3TTRAT]}{dt} = (k_{37f}[RaI1I2I3TTRA][TRAF6] - k_{37r}[RaI1I2I3TTRAT])$$

63) Temporal variation in the concentration of the complex Ra-I1-I2-I3-TRAM-TRIF-TRAF6:

$$\frac{d[RaI1I2I3TTT]}{dt} = (k_{38f}[RaI1I2I3TT][TRAF6] - k_{38r}[RaI1I2I3TTT])$$

64) Temporal variation in the concentration of the complex Ra-I1-I2-I3-TRAM-TRIF-TBK1:

$$\frac{d[RaI1I2I3TTTB]}{dt} = (k_{39f}[RaI1I2I3TT][TBK1] - k_{39r}[RaI1I2I3TTTB])$$

65) Temporal variation in the concentration of the reaction species TBK1:

$$\frac{d[TBK1]}{dt} = -(k_{39f}[RaI1I2I3TT][TBK1] - k_{39r}[RaI1I2I3TTTB])$$

66) Temporal variation in the concentration of the reaction species IRF1:

$$\frac{d[IRF1]}{dt} = -(k_{40f}[RaI1I2I3TTTB][IRF1] - k_{40r}[RaI1I2I3TTTBI]) + (k_{41}[IRFp^*])$$

67) Temporal variation in the concentration of the complex Ra-I1-I2-I3-TRAM-TRIF-TBK1-IRF1:

$$\frac{d[RaI1I2I3TTTBI]}{dt} = (k_{40f}[RaI1I2I3TTTB][IRF1] - k_{40r}[RaI1I2I3TTTBI]) + (k_{40cat}[RaI1I2I3TTTBI])$$

68) Temporal variation in the concentration of the reaction species IRF1p*:

$$\frac{d[IRFp^*]}{dt} = (k_{41}[IRFp^*]) - (k_{41}[IRFp^*]) - 2(k_{42f}[IRFp^*]^2 - k_{42r}[IRFp_2^*])$$

69) Temporal variation in the concentration of the reaction species IRF1p$_2^*$:

$$\frac{d[IRFp_2^*]}{dt} = 2(k_{42f}[IRFp^*]^2 - k_{42r}[IRFp_2^*]) - (k_{43f}[IRFp_2^*] - k_{43r}[IRFp_2^*n])$$

70) Temporal variation in the concentration of the reaction species IRF1p$_2^*$n:

$$\frac{d[IRFp_2^*n]}{dt} = (k_{43f}[IRFp_2^*] - k_{43r}[IRFp_2^*n])$$

71) Temporal variation in the concentration of the reaction species AP1n:

$$\frac{d[AP1n]}{dt} = \\ -(k_{44f}[JNKp{*}n][AP1n] - k_{44r}[JNKp{*}n - AP1n]) - \\ (k_{45f}[P38p{*}n][AP1n] - k_{45r}[P38p{*}n - AP1n]) - \\ (k_{46f}[ERKp{*}n][AP1n] - k_{46r}[ERKp{*}n - AP1n])$$

72) Temporal variation in the concentration of the complex JNKp_2^*n-AP1n:

$$\frac{d[JNKp^*n - AP1n]}{dt} = (k_{44f}[JNKp^*n][AP1n] - k_{44r}[JNKp^*n - AP1n])$$

73) Temporal variation in the concentration of the complex P38p_2^*n-AP1n:

$$\frac{d[P38p^*n - AP1n]}{dt} = (k_{45f}[P38p^*n][AP1n] - k_{45r}[P38p^*n - AP1n])$$

74) Temporal variation in the concentration of the complex ERKp_2^*n-AP1n:

$$\frac{d[ERKp^*n - AP1n]}{dt} = (k_{46f}[ERKp{*}n][AP1n] - k_{46r}[ERKp{*}n - AP1n])$$

75) Temporal expression in the transcriptional readout *Tnf*-α:

$$\frac{d[Tnf\alpha]}{dt} = \\ TmaxTnf * \rho Tnf \left[\frac{\alpha_1 \left(\frac{[AP1n]^{VA1}}{K\beta1^{VA1} + [AP1n]^{VA1}}\right) + \beta_1 \left(\frac{[NF\kappa Bn]^{VB1}}{K\beta1^{VB1} + [NF\kappa Bn]]^{VB1}}\right)}{1 + \alpha_1 \left(\frac{[AP1n]^{VA1}}{K\beta1^{VA1} + [AP1n]^{VA1}}\right) + \beta_1 \left(\frac{[NF\kappa Bn]^{VB1}}{K\beta1^{VB1} + [NF\kappa Bn]]^{VB1}}\right)} \right] - k_d Tnf[Tnf\alpha]$$

76) Temporal expression in the transcriptional readout *Cxcl10*:

$$\frac{d[Cxcl10]}{dt} = \\ TmaxCxc * \rho Cxc \left[\frac{\alpha_2 \left(\frac{[IRF_2n]^{VA2}}{K\beta2^{VA2} + [IRF_2n]^{VA2}}\right) + \beta_2 \left(\frac{[NF\kappa Bn]^{VB2}}{K\beta2^{VB2} + [NF\kappa Bn]]^{VB2}}\right)}{1 + \alpha_2 \left(\frac{[IRF_2n]^{VA2}}{K\beta2^{VA2} + [IRF_2n]^{VA2}}\right) + \beta_2 \left(\frac{[NF\kappa Bn]^{VB2}}{K\beta2^{VB2} + [NF\kappa Bn]]^{VB2}}\right)} \right] - k_d Cxc[Cxcl10]$$

Importantly, two major assumptions underly our mathematical representation of the biochemical reaction mechanism:

a The network model is assumed to represent a "well stirred" reaction system embedded within a spatial homogeneous cell environment.

b The reaction variables (molecular concentrations) are assumed to be continuous functions of time, which is valid only under the assumption that the number of molecules of each species in the reaction volume is sufficiently large.

In general, in the context of our dynamical model, it is more appropriate to think of the trajectories displayed by the signal transduction system as being representative of the average dynamics of the network over an ensemble of cells (i.e. a macrophage culture).
